# Thermodynamics-Informed Machine Learning of Organic Electrode Material Solubility in Nonaqueous Electrolytes

**DOI:** 10.1021/acs.jpcb.6c01822

**Published:** 2026-05-17

**Authors:** Abigail M. Houser, Madhav R. Muthyala, Farshud Sorourifar, Justin Xu, Joel A. Paulson, Shiyu Zhang

**Affiliations:** † Department of Chemistry & Biochemistry, 2647 The Ohio State University, 100 West 18th Avenue, Columbus, Ohio 43210, United States; ‡ Department of Chemical and Biological Engineering, 5228 University of Wisconsin-Madison, 1415 Engineering Drive, Madison, Wisconsin 53706, United States

## Abstract

Organic electrode materials (OEMs) based on abundant elements, such as C, N, O, and S, represent a promising class of sustainable alternatives to traditional transition-metal-based cathodes. However, discovery of new OEMs remains slow and resource-intensive because the design process is dominated by empirical trial-and-error. Although recent machine-learning approaches have accelerated the identification of redox-active compounds, the prediction of OEM solubility—a critical property governing cycle life—remains a major bottleneck. Here, we developed a physics-informed model for predicting OEM solubility using solvation energies (Δ*G*_*solv*_) and sublimation (Δ*G*_*sub*_) energetics derived from a curated set of 44 redox-active organic compounds. While the DFT-calculated Δ*G*_*solv*_ and sublimation enthalpies (Δ*H*_*sub*_) correlate well with experimental measurements, the computed sublimation entropies (Δ*S*_*sub*_) exhibit large systematic errors, thereby limiting the overall predictive accuracy based on first-principles thermodynamics alone. To overcome this limitation, we integrated the physically meaningful computed descriptors into our recently developed symbolic regression framework, SyMANTIC, which learns a corrective model for solubility prediction in nonaqueous electrolytes. Despite the modest data set size, the resulting hybrid model shows good predictive performance under cross-validation and on held-out test compounds, illustrating that this framework can recover compact, chemically meaningful structure–property relationships and offers a new path toward rational design of low-solubility OEMs with reduced reliance on trial-and-error experimentation.

## Graphical abstract

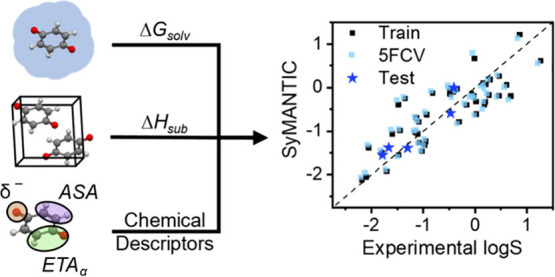

## Introduction

Organic electrode materials (OEMs), containing C, N, O, S, etc., are promising alternatives to transition-metal-based cathode materials due to their abundance, synthetic tunability, and structural diversity.^1,2^ The virtually unlimited design space of organic compounds allows for optimization of cycling stability, solubility, redox potential, etc., for both redox flow battery and solid-state battery applications.^3–5^ Despite these advantages, the development of OEMs still largely relies on a trial-and-error approach, as the correlation between molecular structure and electrochemical performance is nonintuitive. Structural modifications, such as changes in functional groups, can simultaneously alter intermolecular interactions, solid-state packing, and solvation energy, which influence cycling performance in multiple, interconnected ways and frequently introduce trade-offs among key battery performance metrics, such as cycling stability, redox potentials, and solubility. The inherently multiobjective nature of the optimization process, coupled with the need to balance cost and performance, makes the rational design of OEMs particularly challenging. As a result, it is imperative to develop reliable models that correlate molecular structure with key performance metrics.

One promising strategy of this kind is to combine multiobjective machine learning (ML) with computational models that can accurately estimate redox potential, solubility, and synthetic accessibility.^6–12^ Recently, we demonstrated the use of a multiobjective ML algorithm (which we referred to as SPARKLE) to balance competing design criteria to identify promising candidates from large molecular libraries and ultimately streamline the discovery of OEMs with optimized performance.^7^ Sowndarya et al. reported a molecular optimization framework that models radical stability, redox potential, and ease of synthesizability for redox flow battery application.^13^ Pan et al. reported an AI-based approach to design electrolyte additives based on the prediction of redox potential, water solubility, and adsorption energies on the Zn(002) surface.^14^

To effectively utilize ML models for discovery of novel OEMs, accurate predictions of solubility (*S*) are critical.^7,14^ In the context of solid-state batteries, dissolution of OEMs in electrolyte can result in redox shuttle, self-discharge, and ultimately poor cycling performance. Solubility prediction of organic molecules has long been a central challenge in organic chemistry, given its importance in determining drug absorption, bioavailability, and therapeutic effectiveness.^15^ Unfortunately, experimental measurement of solubility is both time-intensive (>2 h per data point) and highly method-dependent, with substantial variability across techniques such as the shake-flask method, slow-stir method, potentiometric titration, UV–vis spectroscopy, NMR, etc.

Early work by Yalkowsky et al. introduced the General Solubility Equation (GSE), which estimates the aqueous solubility of organic compounds with a root-mean-square error (RMSE) of 0.764 log units.^16,17^ The GSE is based on an empirical correlation between solubility, melting point, and the octanol–water partition coefficient (log*P*). While simple and broadly applicable, the GSE and related fragment-based models suffer from low prediction accuracy.^18^ Recently, efforts have been made to predict solubility using the free energy of the dissolution process (eq 1, Figure 1A).

1 $$\Delta G_{sol}=\Delta G_{sub}+\Delta G_{solv}=-2.303\mathrm{RT}\log\left(S_{0}V_{m}\right)$$

**Figure 1:**
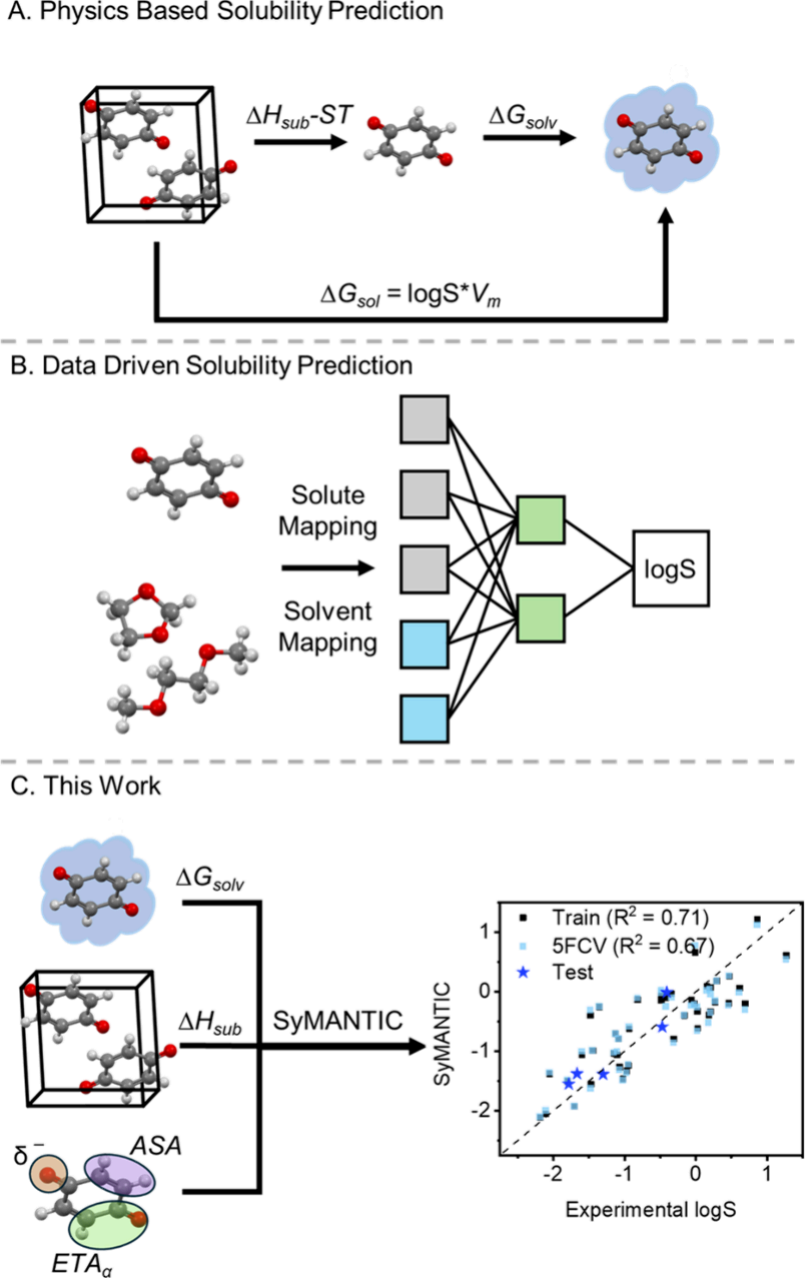
Methods of predicting solubility using (A) the thermodynamic cycle of solution, (B) data-driven (black-box) machine learning methods, and (C) a hybrid model that combines physically meaningful parameters using symbolic regression with SyMANTIC.

This equation relates the free energy of solubility (Δ*G*_*sol*_), which can be converted to the intrinsic solubility (*S*_*0*_) and molar volume (*V*_*m*_), to the sum of sublimation energy (Δ*G*_*sub*_) and solvation energy (Δ*G*_*solv*_). The appeal of this approach is that both Δ*G*_*sub*_ and Δ*G*_*solv*_ can, in principle, be obtained from first-principles calculations, thereby enabling direct estimation of intrinsic solubility. In practice, however, while Δ*G*_*solv*_ can be calculated relatively easily, accurate computation of Δ*G*_*sub*_ is far more demanding and requires a known crystal structure.

Since Δ*G*_*sub*_ is challenging to calculate, recent work has focused on predicting solubility with Δ*G*_*solv*_ alone, without explicitly computing Δ*G*_*sub*_. Although this approach deviates from the thermodynamic cycle of dissolution (Figure 1B), the integration of molecular descriptors with machine learning has shown promising results. For example, Green et al. developed ML models that combine Δ*G*_*solv*_ with molecular descriptors such as *V*_*m*_ and solvent-accessible surface area (SASA) to predict solubility across a range of solvents.^19–21^ The best model shows an RMSE of 0.22 and predicts the solubility of 99.3% of the molecules within log*S* ± 1.

Despite these advancements, it remains an open question whether solubility prediction can be improved by fully incorporating the physical process of dissolution, including both Δ*G*_*solv*_ and Δ*G*_*sub*_.^22–24^ An advantage of such physics-based approaches is that they could, in principle, predict solubility for novel compounds without relying on training data—in a true “zero-shot” fashion. Furthermore, such a physics-based model could yield valuable chemical and thermodynamic insights, such as enthalpic and entropic contributions to solubility, which are useful for rational design.^15^

Herein, we explore whether it is possible to build a solubility model for OEMs in nonaqueous electrolytes by explicitly considering both Δ*G*_*solv*_ and Δ*G*_*sub*_ (Figure 1C). Applying this approach, which has largely been developed for predicting drug solubility, to OEMs introduces several key considerations.^22–24^ First, unlike pharmaceutical compounds that are typically aliphatic, OEMs are primarily aromatic, necessitating the construction of a new testing set tailored to OEM-like compounds. Second, previous studies on the solubility of pharmaceutical compounds have generally focused on aqueous solubility prediction, whereas applying this approach to nonaqueous environments requires modeling of the solvation process in nonaqueous solvents. Finally, because OEMs are used in salt-containing electrolytes, it is important to compare solubility in both pure/neat solvents and salt-containing electrolyte solutions.

To address these differences, we built a data set of experimentally measured solubilities for redox-active organic compounds, and calculated their Δ*G*_*solv*_, enthalpy of sublimation (Δ*H*_*sub*_), and entropy of sublimation (Δ*S*_*sub*_).^25^ To capture the influence of salts, solubilities were measured in both neat solvents and electrolyte solutions (solvent + salt) using a highly efficient cyclic voltammetry (CV) method. We then analyzed the theoretical limitations of this physics-based framework. While Δ*G*_*solv*_ and Δ*H*_*sub*_ can be calculated with reasonable accuracy using first-principles methods, the calculation of Δ*S*_*sub*_ remains poor, resulting in weak overall correlation with experimental solubility. To address this issue, we apply symbolic regression using our recently developed SyMANTIC framework to build an interpretable solubility model incorporating Δ*G*_*solv*_, Δ*H*_*sub*_, and a small set of Mordred (molecular) descriptors.^26^ Upon performing 5-fold cross-validation, the model achieves a mean RMSE of 0.46 ± 0.14 log*S* units. Compared with higher-capacity black-box ML models, which can suffer from overfitting issues on small data sets, our learned SyMANTIC model displays generalizable and chemically meaningful trends.

## Methods

### General Experimental Details

All experiments were carried out at ambient temperature and pressure unless otherwise stated. Unless otherwise noted, all solvents were degassed with nitrogen and used directly. All glassware was dried at 80 °C before use. UV–Vis spectra were recorded on a Cary 60 spectrometer equipped with a Unisoku UnispeKs cryostat. Cyclic voltammetry (CV) measurements were performed at room temperature under ambient atmosphere using Bio–Logic SAS SP–50 potentiostat with a glassy carbon working electrode, a platinum wire counter electrode, and a Ag/AgNO_3_ (0.01 M) reference electrode. Lithium bis­(trifluoromethanesulfonyl)­imide (97% purity) was obtained from Combi–Blocks. Lithium nitrate (99.99% purity, trace metals), 1,3-dioxolane (99.8% purity, anhydrous, ∼75 ppm BHT as inhibitor) were obtained from Sigma-Aldrich. 1,2-Dimethoxyethane (>99% purity, anhydrous) was obtained from Chem–Impex Intl. Inc. All redox-active molecules used in solubility tests were acquired from reputable suppliers and utilized without further purification.

### Solubility Measurements

Calibration curves were established using measurements obtained by CV analysis.^27^ Redox-active compounds of a certain weight (typically 0.5–2 mg) were dissolved in the electrolyte solution (2.0 mL), composed of a 1:1 dimethoxyethane:1,3-dioxolane (DOL:DME) solution of 1 M LiTFSI and 0.2 M LiNO_3_ to obtain a homogeneous solution. The electrolyte was made in the glovebox under argon atmosphere at the beginning of each day to prevent excessively high concentrations of water. The solution was transferred to an oven-dried CV vial, sparged with nitrogen flow for 30 s, and a CV was measured under ambient air at room temperature with a scan rate of 100 mV/s. We note that excessive N_2_ purging can lead to solvent loss due to the volatility of the DOL:DME mixture, which may introduce inaccuracies in the measured concentrations. After purging, six 0.5 mL aliquots of the 1:1 DOL:DME electrolyte (1 M LiTFSI and 0.2 M LiNO_3_) were sequentially added to the CV vial, and a new CV scan was collected after each addition. The current response of the first reduction peak was quantified using the baseline analysis tool in EC–Lab (v11.26). These current values were then plotted against the corresponding analyte concentrations to generate calibration curves. To ensure reliability, only calibration curves with a correlation coefficient (*R* ^2^) greater than 0.97 were used for subsequent analysis. Saturated solutions of redox-active compounds were prepared by sonicating the analyte (20–50 mg) in 1:1 DOL:DME electrolyte (1 M LiTFSI and 0.2 M LiNO_3_, 0.5 mL) for 10 min. If the mixture became homogeneous, additional analyte was added until saturation was reached. The resulting suspension was then filtered through a glass fiber plug, and an aliquot was diluted for CV analysis. The solubility of the analyte was determined using the calibration curves described above (see Supporting Information, Table S1).

### Lattice Energy Calculations

Using Quantum Espresso 6.7, periodic density functional theory (DFT) geometry optimization calculations were used to determine the extended lattice energy.^28,29^ These calculations used a plane wave energy cut off of 60 Ryd and a mixing beta rate of 0.5, as discussed in Cersonsky et al.^30,31^ The energy and force convergence thresholds were set to 1.0E–4 Ryd and 1.0E–3 Ryd/Bohr, respectively, again per Cersonsky et al. Additionally, the calculations utilized ultrasoft pseudopotentials with GIPAW reconstruction from pslibrary for each atom.^32,33^ The calculations were performed using the PBE exchange-correlation functional with the Grimme D2 dispersion correction.^34,35^ After analysis of different parameters, K-points for the extended lattice calculations were chosen to be 30 divided by the lattice parameters of each crystal structure’s unit cell. These were generated automatically in a Monkhorst–Pack grid with no offset.^36^ A second calculation was performed for each of the structures analyzed. Isolation of the molecule from the crystal structure was performed using the same parameters, with a unit cell size four times the initial size to ensure minimal intermolecular interactions. The crystal structure coordinates were adjusted by shifting all atoms by a specified amount. This was done to not disrupt bonding within a molecule, as this could drastically change the final energy. Using these two calculations, the lattice energy was determined by the summation of the isolated energy (*E*_*mol*_) for each of the molecules in the unit cell and subtracting this from the total energy of the extended crystal lattice (*E*_*cryst*_) (eq 2).

2 $$U_{latt}=E_{cryst}-\Sigma E_{mol}$$

Additional details can be found in the Supporting Information.

### Solvation Energy Calculations

All calculations were performed using Gaussian 16. The geometry optimizations were done using the B3LYP–D3 method with 6–311+G­(d,p) basis set.^37–39^ To confirm that the optimized structures are true stationary points, harmonic vibrational frequency analysis was performed, and no imaginary frequencies were obtained. *ΔG*_*solv*_ for the molecules were calculated using their gas phase free energy as well as the free energy calculated in a solvated environment using a SMD model of DOL:DME (see Supporting Information).^40^

### Sublimation Entropy Calculations

*ΔS*_*rot*_ and *ΔS*_*trans*_ were calculated using gas-phase DFT geometry optimization as detailed previously. Due to the computational expense of phonon calculations, *ΔS*_*vib*_ was estimated using a set of experimental entropy terms reported in McDonagh et al.^24^

### General Modeling Details

All computational modeling and statistical analysis were performed using Python 3.11 with key libraries including scikit-learn (linear regression analysis & random forest) and SyMANTIC (for symbolic regression).

### Preprocessing Solubility Data

All models were trained on the same data set of *N* = 44 organic molecules. For each molecule collected, we collected log*S* (experimentally curated solubility), a set of quantum descriptors (*ΔG*_*solv*_, *ΔH*_*sub*_, etc.), and a large set of Mordred descriptors that are calculated from SMILES representation of molecules using the Mordred package.^41^ These descriptors were concatenated with the original features to form an extended design matrix. Non-numeric descriptor columns are dropped, and zero-variance features were removed using the empirical variance across the 44 molecules.

## Results and Discussion

### Solubility Measurements of Redox-Active Organic Compounds with Cyclic Voltammetry

OEM-like molecules typically contain polyaromatic structures designed to have low HOMO–LUMO gaps for conductivity and π–π stacking for low solubility. Therefore, they often exhibit very low solubility that is difficult to measure with traditional methods.^18,42–45^

Our lab previously developed a UV–vis method to measure the solubility of a series of anthraquinones.^6^ While this method was sufficient for a data set of 30 compounds, it is too time-consuming for larger studies (ca. 2 h per measurement). Additionally, due to the low solubility of OEMs in the desired electrolyte, the Beer’s law calibration curve must be constructed in a more polar solvent, assuming that the molar extinction coefficient of the OEM-like molecule remains unchanged—an assumption that is not always valid. To address these limitations, we explored whether CV could serve as an alternative approach for measuring the solubility of redox-active organic molecules.

There has been one report of solubility measurement using CV by Pastore et al.^27^ This method assumes a linear correlation between the CV peak current response (*i*_*p*_) and analyte concentration (*C*) based on the Randles–Ševčík eq (eq 3), given that the scan rate (*v*), temperature, electrode area (*A*), number of electrons per redox event (*n*), and diffusion coefficient (*D*) remain constant.

ip=2.69×105n3/2ACDvat25C° 3

Taking advantage of the redox-active nature of OEM-like compounds, we applied this method by first constructing a calibration curve in the electrolyte of interest—typically a battery electrolyte such as 1:1 DOL:DME containing 1 M LiTFSI and 0.2 M LiNO_3_—using solutions with known analyte concentrations. Subsequent CV analysis of an aliquot taken from the saturated sample enables accurate determination of maximum solubility from the current response (see Supporting Information, Figure S5 for details). This method is significantly faster than the UV–vis approach (ca. 30 min per measurement) and avoids the aforementioned limitations associated with UV–vis analysis.

### Comparison of Solubility in Pure Solvent vs Electrolyte (Solvent and Salt Mixture)

While conventional first-principles solubility calculations focus on predicting drug solubility in pure aqueous environments, the goal of our study is to establish the solubility of organic compounds in nonaqueous electrolytes, where both solvent and supporting salts (often lithium salts) are present. Most Δ*G*_*solv*_ models in the literature do not account for the effect of salt on solubility.

To understand the effect of Li salts on the solubility of redox-active organic compounds, we performed a comparative study by using the same set of 10 anthraquinones. The solubility of each compound in pure 1:1 DOL:DME was measured and compared to the solubility when using a 1:1 DOL:DME electrolyte (1 M LiTFSI and 0.2 M LiNO_3_) (Figure 2).

**Figure 2:**
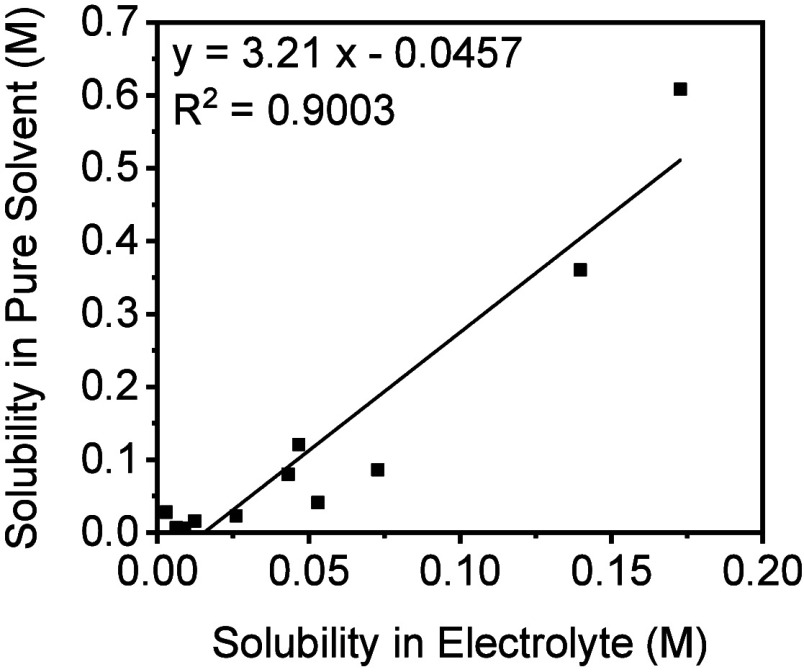
Correlation of anthraquinone solubilities in 1:1 DOL:DME electrolyte (1 M LiTFSI and 0.2 M LiNO_3_) and pure 1:1 DOL:DME using the CV method.

For most of the measured anthraquinones, solubility in pure 1:1 DOL:DME was approximately three times higher than in the electrolyte containing Li salts. The difference in solubility between the neat solvent and the electrolyte was less pronounced for poorly soluble compounds (<0.05 M), such as anthraquinone–2-carboxylic acid, 1,2-diaminoanthraquinone, and 2-aminoanthraquinone. Nevertheless, the solubilities with and without salts correlate well (*R* ^2^ = 0.90), indicating that although the presence of salts affects the absolute solubility of OEM-like compounds, the general trend and relative ranking remain consistent. This analysis supports our approach of using computationally derived Δ*G*_*sub*_ and Δ*G*_*solv*_ values (without explicitly including salt effects) to predict or rank the solubility of redox-active organic molecules in electrolytes. In other words, models built using Δ*G*_*solv*_ in pure solvent, combined with Δ*G*_*sub*_, are expected to preserve ranking in the corresponding electrolyte, even if the absolute values differ.

With the validated CV-based solubility measurement, we measured the solubility of 44 redox-active organic molecules in 1:1 DOL:DME electrolyte (1 M LiTFSI and 0.2 M LiNO_3_). These 44 redox-active molecules also have reported crystal structures in the Cambridge Structural Database (CSD),^46,47^ which supports first-principles calculations of Δ*G*_*sub*_. From the collected data, a broad solubility range (0.00660 M–18.5 M) was observed across this diverse set of structures that include typical redox-active functional groups, such as quinones and heteroaromatic motifs (Figure 3, see Supporting Information, Table S1).

**Figure 3:**
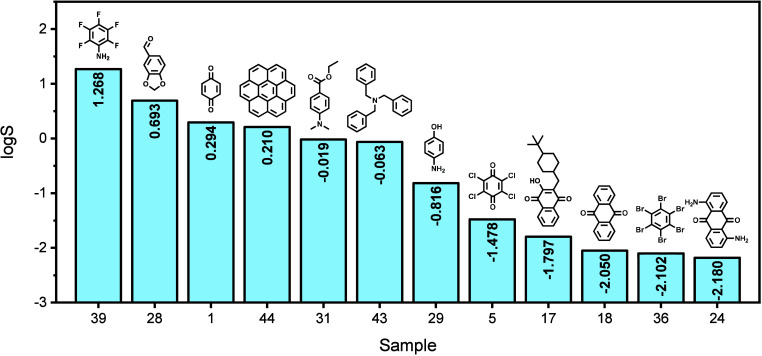
Selected redox-active compounds with sample ID, structure, and log*S* in 1:1 DOL:DME with 1 M LiTFSI and 0.2 M LiNO_3_.

### First-Principles Calculation of Δ*G_solv_* and Δ*G_sub_*

To begin constructing a solubility model, we calculated Δ*G*_*solv*_ and Δ*G*_*sub*_ using state-of-the-art literature methods.^6,22–24,30,48^ Combining Δ*G*_*solv*_ and Δ*G*_*sub*_ enables the prediction of solubility in pure organic solvents, which show a linear correlation with solubility in electrolyte, as demonstrated in the section above. Computation of Δ*G*_*solv*_ is well established, based on the difference between the gas-phase free energy of a molecule and its solvated free energy, modeled using the DFT method. In our case, Δ*G*_*solv*_ was computed at the B3LYP/6–311+G­(d,p) level of theory, with GD3 empirical dispersion corrections and the SMD solvent model using either diethyl ether or 1:1 DOL:DME as the solvent.^6,48^ To validate our computational approach, we benchmarked it against the Minnesota Solvation (MNSOL) Database, comparing calculated Δ*G*_*solv*_ values with experimental data for molecules in diethyl ether.^40^ The method showed strong agreement (*R* ^2^ = 0.85, Figure 4A). After confirming its accuracy, Δ*G*_*solv*_ values were computed for all 44 molecules in our data set using 1:1 DOL:DME as the solvent (see Supporting Information, Table S10).

**Figure 4:**
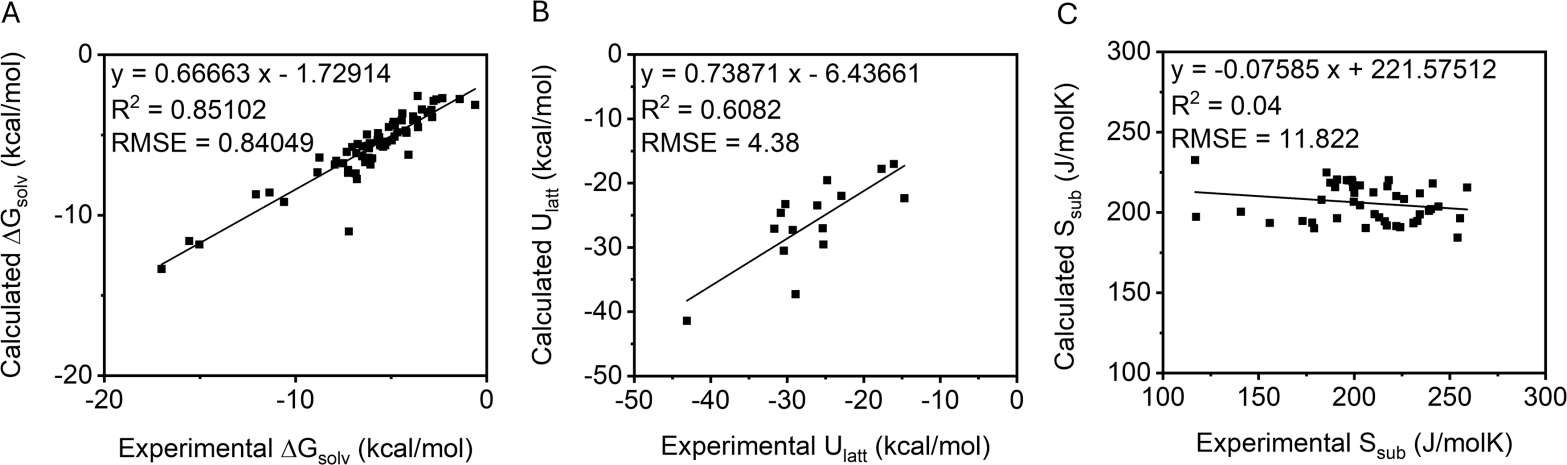
Correlation between calculated and experimental values for (A) Δ*G*_*solv*_ from the Minnesota Solvation (MNSOL) database, (B) *U*_*latt*_ from the NIST database, and (C) Δ*S*_*sub*_ from McDonagh et al. showing the accuracy of each type of calculation.

To calculate Δ*G*_*sub*_, both Δ*H*_*sub*_ and Δ*S*_*sub*_ were computed separately according to the Gibbs free energy relation. For Δ*H*_*sub*_, the lattice energy (*U*_*latt*_) was calculated from the crystal structures of the target molecules, with *U*_*latt*_ correlated to Δ*H*_*sub*_ by an empirical offset of −2RT (eq 4):^25^

4 $$\Delta H_{sub}=-U_{latt}-2\mathrm{RT}$$

Using a periodic DFT approach implemented in Quantum ESPRESSO (see Supporting Information, Table S9), the total crystal energy (*E*_*cryst*_) was obtained from calculations on the full unit cell.^28–31^ A second calculation with a single isolated molecule provided the molecular energy (*E*_*mol*_). The difference between these values, normalized by the number of molecules per unit cell, yields the lattice energy (eq 2).

To validate this approach, a set of compounds with known crystal structures and experimental lattice energies from the NIST database were analyzed.^50,51^ The calculated lattice energies correlated reasonably well with experimental data (*R* ^2^ = 0.61, Figure 4B, also see Supporting Information, Table S8).

Finally, Δ*S*_*sub*_ is needed to relate Δ*H*_*sub*_ to Δ*G*_*sub*_. Unfortunately, experimental Δ*S*_*sub*_ is not commonly reported in the literature. With limited experimental Δ*S*_*sub*_ data, McDonagh et al. attempted to calculate Δ*S*_*sub*_ by computing the vibrational entropy (Δ*S*_*vib*_) of the crystalline phase and the translational and rotational entropies (Δ*S*_*trans*_ and Δ*S*_*rot*_) of the gas phase (eq 5):^24^

5 $$\Delta S_{sub}=\Delta S_{vib}-\left(\Delta S_{trans}+\Delta S_{rot}\right)$$

However, the calculated Δ*S*_*sub*_ values show essentially no correlation with experimental values (*R* ^2^ = 0.05). We repeated the calculations using several different functionals and basis sets, which did not improve the outcome (*R* ^2^ = 0.04, Figure 4C). Though there are methods in the literature to more accurately calculate *ΔS*_*sub*_, we did not explore these due to computational cost.^52–54^ Despite this limitation, we proceeded to incorporate the computed Δ*S*_*sub*_ values into our solubility model, motivated by the precedent of McDonagh et al. in employing entropic terms to predict aqueous solubilities of drug-like molecules. Nevertheless, with the apparent discrepancy between computed and experimental Δ*S*_*sub*_, we anticipated that these inaccuracies would propagate into the overall solubility predictions and degrade model performance. As a result, compensating for this deficiency requires the introduction of nonfirst-principles corrections (*vide infra*).

### Developing a Solubility Model Using Δ*G_solv_* and Δ*H_sub_*

Although both Δ*G*_*solv*_ and Δ*H*_*sub*_ individually exhibited strong correlations with experimental data, the full thermodynamic cycle (incorporating Δ*S*_*sub*_) yielded poor predictive accuracy (Figure 5A). We attributed this discrepancy to substantial error/noise in the calculated Δ*S*_*sub*_ values, as evidenced by Figure 4C. To rigorously test whether this deviation could be mitigated through parametrization, we constructed a linear model by reoptimizing the thermodynamic coefficients using the well-established L-BFGS algorithm.^55^ The resulting model (eq 6; where α = 0.38075, β = 0.107058, γ = 0.057, and δ = 0.540), shown in Figure 5B, yielded an *R* ^2^ of 0.38:

6 $$\log S=\alpha \Delta G_{solv}-\beta \left(\Delta H_{sub}-\Delta S_{sub}T\right)-\gamma \log\left(V_{m}\right)-\delta$$

**Figure 5:**
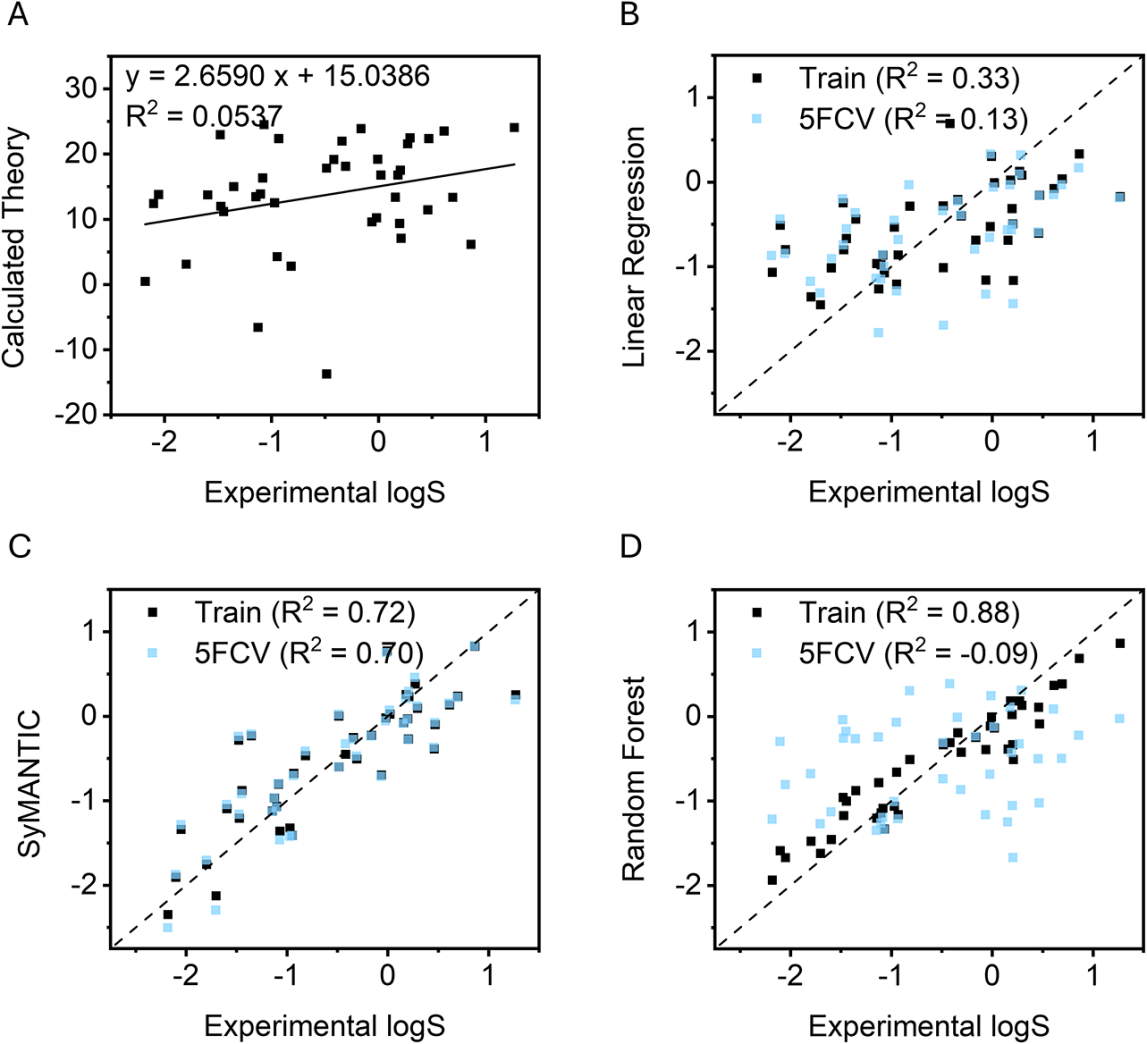
Plots of solubility models using (A) the thermodynamic solubility equation, (B) optimized thermodynamic solubility equation, (C) SyMANTIC method with Mordred descriptors, Δ*G*_*solv*_, and Δ*H*_*sub*_, and (D) a Random Forest black box model (see Supporting Information).

This result suggests that the errors associated with the sublimation entropy term are not adequately corrected by the global coefficient optimization alone.

To overcome the constraints of linear parametrization while preserving interpretability, we transitioned to a symbolic regression approach using our recently developed (open-source) symbolic regression framework, SyMANTIC.^26^ Unlike conventional “black-box” machine learning (ML) algorithms that hide mechanistic details, symbolic regression generates transparent, closed-form analytical expressions that can be examined directly at the descriptor level. The candidate feature space was constructed by combining computed thermodynamic quantities (Δ*G*_*solv*_ and Δ*H*_*sub*_) together with molecular descriptors from the Mordred library that encode features such as size, topology, and electronic character.^41^ In this way, the model can benefit from both physically motivated thermodynamic quantities and broader structure–property patterns that may not fully be captured by the approximate thermodynamic cycle alone.

To develop a predictive model, the SyMANTIC workflow was applied to our full data set of 44 data points. The framework identified a functional form through a multistage optimization procedure. First, important primary descriptors were selected based on their relationship to log*S* using a mutual information-based feature selection approach. These features are then recursively combined using a restricted library of algebraic and simple nonlinear operators (e.g., + , −, ^∧^2, /, *) to generate a structured, high-dimensional expression space. To manage the combinatorial growth, Sure Independence Screening (SIS) is applied to filter the generated candidates, followed by a sequence of $l_{0}$ -regularized regression steps. This sparsity-promoting optimization explicitly penalizes complexity, isolating a minimal subset of “basis functions” that best explain the variance in solubility. The final model converges to the following symbolic eq (eq 7; where ε = 0.3014, ζ = 0.004736, σ = 0.04027, and ψ = 1.55106):

7 $$\log S=\varepsilon \frac{\Delta G_{solv}\left(Xp_{4dv}-Xp_{6dv}\right)}{ETA_{\alpha }}-\zeta \frac{Xp_{7dv}\left(\Delta H_{sub}-ASA\right)}{JGI_{2}}-\sigma \frac{MWC_{07}}{Xp_{6dv}\left(ETA_{\alpha }-ATSC_{3s}\right)}+\psi$$

The descriptors appearing in eq 7 are completely defined in the Supporting Information, where we provide the relevant equations, descriptor-family context, and primary references. Briefly, ASA (Labute Approximate Solvent-Accessible Surface Area) reflects the overall exposed molecular surface; *Xp*_4*dv*_, *Xp*_6*dv*_, and *Xp*_7*dv*_ are valence-weighted Chi path indices that capture aspects of molecular connectivity and topology over different path lengths; *ETA*_*α*_ is an Extended Topochemical Atom descriptor related to the core skeletal/electronic constitution of the molecular graph; *JGI*_2_ is the mean topological charge index of order 2, which captures graph-based charge transfer patterns between atom pairs separated by two bonds; *ATSC*_3*s*_ is a centered Moreau–Broto autocorrelation of lag 3 weighted by intrinsic state and thus captures how the electro-topological behavior is distributed across three-bond neighborhoods; and *MWC*_07_ is the molecular walk count of order 7, which provides a measure of graph complexity associated with size, branching, and cyclicity.^56–62^ Taken together with Δ*G*_*solv*_ and Δ*H*_*sub*_, the resulting expression is best interpreted as a compact descriptor-level surrogate that couples solvation, cohesive penalties, and molecular topology/electronic organization. We therefore do not interpret the individual symbolic terms as standalone mechanistic quantities or literal thermodynamic identities, but rather as learned combinations that capture structure–property trends within the present chemical domain.

By coupling topological and electronic descriptors with thermodynamic variables, the symbolic model can account for systematic variation that is not fully represented in the underlying thermodynamic cycle alone. In particular, the learned correction appears to recover aspects of molecular structure that are associated with solubility but are not captured well by the noisy Δ*S*_*sub*_ term. Importantly, this correction is expressed through an explicit analytical equation rather than an opaque nonlinear mapping, allowing the model to remain “inspectable” at the descriptor level while improving predictive accuracy. We therefore view the learned expression not as a first-principles replacement for the thermodynamic cycle, but as a compact surrogate that augments it with statistically learned structure–property information.

Given the limited data set size (*N* = 44), we used a validation strategy designed to separate structural robustness from fully repeated model discovery. First, SyMANTIC was run on the full data set to identify a sparse symbolic form, yielding a training *R* ^2^ of 0.71 (Figure 5C). Second, we performed a post hoc 5-fold cross-validation (5FCV) in which the symbolic structure was held fixed and only the numerical coefficients were re-estimated each training fold. This protocol serves as a stress test of structural stability; rather than asking whether repeated searches recover the same equation, it asks whether the selected functional form retains predictive utility when it is recalibrated on reduced data. Because the structure was selected using the full training data set, this fixed-structure 5FCV is not a fully unbiased estimate of generalization error. Instead, it probes whether the learned symbolic form reflects stable structure–property relationships rather than simply memorizing a small data set.

Across the five folds, the model maintained a mean *R* ^2^ value of 0.67 (Figure 5C). To further evaluate the stability of the full discovery process, we additionally performed repeated cross-validation in which the entire SyMANTIC workflow (including descriptor selection, symbolic expansion, and model fitting) was rerun within each fold across 5 random seeds; the results can be found in the Supporting Information. As expected, the resulting held-out performance was a bit lower than that of the fixed-structure analysis but remained clearly positive (*R* ^2^ = 0.53, RMSE = 0.35 based on per-sample mean predictions). In addition, a simple feature sensitivity analysis showed that the main ingredients of the final model recur repeatedly across most folds.

To establish a performance benchmark, we trained a random forest (RF) regression model following conditions identical to those used for SyMANTIC. As shown in Figure 5D, the RF model exhibited a clear divergence between training and validation performance; while it achieved a high correlation on the training set (*R* ^2^ = 0.83), it performed poorly under 5FCV (*R* ^2^ = 0.013). This discrepancy highlights the (well-known) susceptibility of black-box ML models to overfitting in small-data regimes, where they can memorize split-specific patterns rather than recover the more transferable structure–property relationships captured here by the sparse symbolic model.

To further assess the practical utility of the model, we predicted the solubility of 20 previously unmeasured compounds, from which 5 compounds were selected based on chemical availability and expert judgment. These compounds were not used in any component of the model’s training process. Subsequent experimental measurements showed good agreement with the predicted solubilities, particularly in the low- to moderate-solubility regime (Figure 5C). Relative to the alternative models considered here, SyMANTIC provided closer agreement between predicted and measured values, supporting the conclusion that the discovered expression captures chemically meaningful trends that retain predictive utility on unseen compounds.

## Conclusion

Physics-based prediction of solubility for organic electrode materials (OEMs) in nonaqueous electrolytes remains challenging. Most existing thermodynamic and data-driven models have been developed for aqueous systems, so organic solubility in battery-relevant (nonaqueous) media is comparatively understudied. A key bottleneck is the scarcity of reliable experimental solubility data for nonaqueous systems. In this work, by using an efficient CV protocol, we constructed a curated data set of solubilities for redox-active organic molecules in Li-salt-containing DOL:DME electrolytes. For this set, we computed thermodynamic quantities for the dissolution process and showed that Δ*G*_*solv*_ and Δ*H*_*sub*_ correlate reasonably well with experiment, whereas Δ*S*_*sub*_ exhibits large systematic errors that severely limit purely first-principles solubility predictions.

We addressed this limitation by combining physics-based descriptors with our recently developed sparse symbolic regression framework (SyMANTIC). By integrating physically meaningful computed quantities (such as Δ*G*_*solv*_ and Δ*H*_*sub*_) with a small number of chemically interpretable Mordred molecular descriptors, the resulting hybrid model learns a corrective relationship for solubility prediction in nonaqueous electrolytes. Despite the modest data set size, this model shows strong cross-validated performance, which indicates that the learned relationship can generalize beyond the training set, offering a scalable path toward rational design of low-solubility OEMs while reducing reliance on trial-and-error experimentation. More broadly, this work highlights how physics-informed symbolic regression can compensate for systematic deficiencies in specific thermodynamic terms while retaining interpretability and chemical insight.

## Supplementary Material



## Notes

Experimental details, including characterization data, spectra, computational procedures, and results (PDF)
