# Supplementary material for: Thermodynamics-Informed Machine Learning of Organic Electrode Material Solubility in Nonaqueous Electrolytes
Source: J Phys Chem B. 2026 May 17;130(21):5378–86. doi: 10.1021/acs.jpcb.6c01822 (PMC13224181; doi:10.1021/acs.jpcb.6c01822)
Supplement: Supplementary file 1 [file jp6c01822_si_001.pdf]

Electronic Supporting Information for:

Thermodynamics-Informed Machine Learning of Organic Electrode Material Solubility in Nonaqueous Electrolytes

Abigail M. Houser,[a] Madhav R. Muthyala,[b] Farshud Sorourifar,[b] Justin Xu,[a] Joel Paulson,*[b] and Shiyu Zhang*[a]

[a] Department of Chemistry and Biochemistry, The Ohio State University, 100 W. 18th Avenue, Columbus, Ohio 43210, United States.

[b] Department of Chemical and Biological Engineering, University of Wisconsin–Madison, 1415 Engineering Drive, Madison, Wisconsin 53706, United States.

* Correspondence: zhang.8941@osu.edu, joel.paulson@wisc.edu

Contents

Cyclic Voltammetry Study Details 2
Determination of the solubility of redox–active compounds in 1:1 DOL:DME electrolyte (1 M LiTFSI and 0.2 M $LiNO_3$) 2
Measurement of diffusion coefficient as a function of concentration 6
Variable time point study of solubility measurements with CV methods 7
Determination of the solubility of redox–active compounds in pure 1:1 DOL:DME 7
Comparison of solubility measurements with UV–vis and CV methods 8
Dataset Construction for Lattice Energy Calculations 9
Lattice Energy Calculation using Periodic DFT 9
Selection of K-point mesh and expansion size for lattice energy calculations 9
Method of lattice energy calculations for quinone with known crystal structures 11
Details of lattice energy calculation 12
Calculation of sublimation enthalpy from lattice energy 13
DFT Computational Details 13
Sublimation entropy computations 13
Solvation energy computations 14
Calculation of Additional Solubility Descriptors 16
Data–Driven Solubility Modeling 17
Linear regression models derived from the thermodynamic equation of solution 17
Symbolic regression model with SyMANTIC 18
Random Forest regression model baseline 19
Determination of additional equations to model solubility 20
Interpretability of SyMANTIC Model 20
10-Fold Cross-Validation and Descriptor Sensitivity Analysis of end-to-end SyMANTIC workflow 22
References 25
Gaussian 16 Citation 26

Cyclic Voltammetry Study Details

Determination of the solubility of redox–active compounds in 1:1 DOL:DME electrolyte (1 M LiTFSI and 0.2 M $LiNO_3$)

For a set of 13 compounds, the solubility measurement was repeated twice more to determine the accuracy of these measurements. One of these measurements (trial 3) was performed in an argon glove box to verify that measurements outside of the box are valid. The standard deviation of these measurements is seen in Fig. S2 and Table S2.

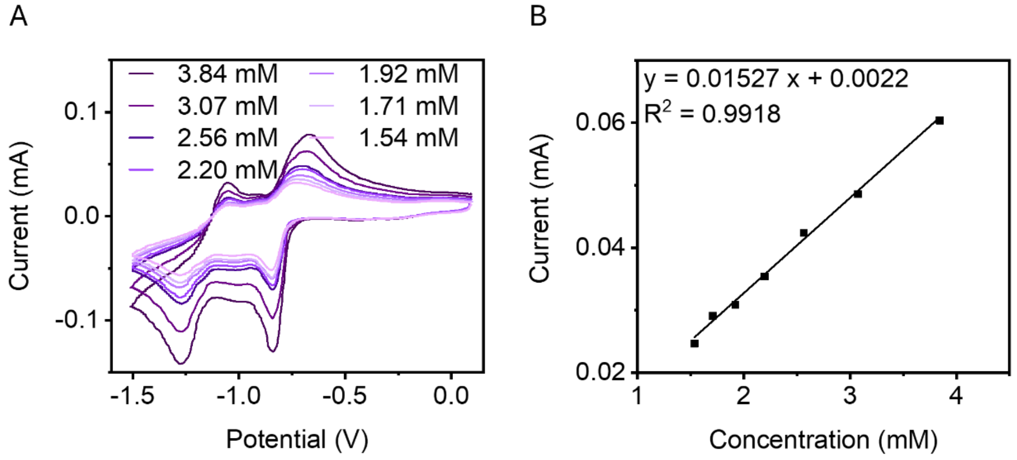

Fig. S1. Example of (A) CV measurements and (B) calibration curve used to determine the concentration of acenaphthenequinone in 1:1 DOL:DME electrolyte (1 M LiTFSI and 0.2 M $LiNO_3$).

Table S1. Experimental solubility of redox–active compounds in 1:1 DOL:DME electrolyte (1 M LiTFSI and 0.2 M $LiNO_3$).

ID	Crystal Name	Compound Name	SMILES	Solubility (M)	R^2 of Calibration Curve
**Quinones**					
25	ACNAQU	Acenaphthenequinone	C1(=O)C(=O)C2=[C][C]=[C]C3=[C][C]=[C]C1=C23	0.03367	0.9918
18	ANTQUO	Anthraquinone	c1cc2c(cc1)C(=O)c1c(C2=O)cccc1	0.00891	0.9847
1	BNZQUI	p–Benzoquinone	C1=CC(=O)C=CC1=O	1.96697	0.9871
24	BOSMUS	1,5–Diaminoanthracene–9,10–dione	Nc1cccc2C(=O)c3c(N)cccc3C(=O)c12	0.0066	0.9847
21	BRANTO	Alpha–bromoanthraquinone	BrC1=[C][C]=[C]C2=C1C(=O)C1=C(C2=O)[C][C][C][C]1	0.07899	0.9983

15	BRNAPQ	2–Bromo–1,4–naphthoquinone	BrC1=[C]C(=O)C2=C(C1=O)[C]=[C][C]=[C]2	1.59704	0.9975
3	BZQDCL10	2,5–Dichloro–1,4–benzoquinone	ClC1=CC(=O)C(=CC1=O)Cl	0.45473	0.9936
20	CANTRQ01	1,5–Dichloroanthraquinone	C1(=C2C(=[C][C]=[C]1)C(=O)C1=C([C]=[C][C]=C1C2=O)Cl)Cl	0.02536	0.9823
4	CLANAC	Chloranilic acid	ClC1=C(O)C(=O)C(=C(C1=O)O)Cl	0.04432	0.9937
19	CLANTO	alpha–Chloroanthraquinone	ClC1=[C][C]=[C]C2=C1C(=O)C1=[C][C]=[C][C]=C1C2=O	0.08263	0.9849
2	CLBENQ	Chloro–1,4–benzoquinone	ClC1=CC(=O)C=CC1=O	4.09440	0.9788
23	DHANTQ01	1,5–Dihydroxy–9,10–anthraquinone	c1(cccc2c1C(=O)c1c(C2=O)c(ccc1)[O])[O]	0.01982	0.9928
7	DHXBZQ	2,5–Dihydroxy–1,4–benzoquinone	O=C1C(=CC(=O)C(=C1)O)O	0.11676	0.9743
9	GUFMAV	2,3,5,6–Tetrafluorohydroquinone	Oc1c(F)c(c(O)c(c1F)F)F	7.30489	0.9737
12	GUFMEZ	2,3,5,6–Tetrabromohydroquinone	Oc1c(Br)c(Br)c(O)c(c1Br)Br	0.1069	0.9874
13	HIQTEG	3,5–Di–t–butyl–1,2–benzoquinone	O=C1C(=O)C(=CC(=C1)C(C)(C)C)C(C)(C)C	1.43518	0.9982
10	IBOVAZ	2,3,5,6–tetraamino–1,4–benzoquinone	O=C1C(=C(N)C(=O)C(=C1N)N)N	0.32759	0.9934
11	JOYQUJ01	2,5-Dibromohydroquinone	Brc1c(O)cc(Br)c(c1)O	1.0567	0.9968
22	JUNBIF	1–hydroxy–9,10–anthraquinone	O=C1c2c(O)cccc2C(=O)c2ccccc12	0.08512	0.998
14	NAPHQU	1,4–Naphthoquinone	C1(=O)[C]=[C]C(=O)C2=[C][C]=[C][C]=C12	0.32690	0.9897
26	SNQUOX	syn–1,2–Naphthoquinone–1–oxime	C\1(=N/O)/C(=O)C=Cc2ccccc12	0.49203	0.9959
5	TCBENQ	Tetrachloro–p–benzoquinone	ClC1=C(Cl)C(=O)C(=C(C1=O)Cl)Cl	0.03323	0.9976
6	TCLOBQ	Tetrachloro–o–benzoquinone	ClC1=C(Cl)C(=C(Cl)C(=O)C1=O)Cl	2.94808	0.9852
8	TFBENQ	Tetrafluoro–p–benzoquinone	C1(=O)C(=C(C(=O)C(=C1F)F)F)F	0.98243	0.9895
17	UHUKAJ	2–((4–(1,1–Dimethylethyl)cyclohexyl)methyl)–3–hydroxy–1,4–naphthalenedione	C1(=O)C(=C(C(=O)c2ccccc12)C[C@@H]1CC[C@H](CC1)C(C)(C)C)O	0.01596	0.9778

16	UPAGUO	2–Methoxynaphthalene–1,4–dione	O(C1=CC(=O)c2c(C1=O)cccc2)C	0.03574	0.9963
**Non–Quinones**					
29	AMPHOL01	p–Aminophenol	Nc1ccc(O)cc1	0.15277	0.9994
33	AZOBEN01	trans-Azobenzene	c1ccc(cc1)N=Nc2ccccc2	0.3906	0.9878
44	CORONE	Coronene	c1cc2ccc3ccc4ccc5ccc6ccc1c7c2c3c4c5c67	1.62115	0.9842
31	DUNHIE	Ethyl 4–(dimethylamino)benzoate	CCOC(=O)c1ccc(cc1)N(C)C	0.95612	0.9955
30	FEVBIS	2–Cyano–3–(4–hydroxyphenyl) propenoic acid	OC(=O)C(=Cc1ccc(O)cc1)C#N	0.07490	0.9944
28	FOMZUD	Piperonal	O=Cc1ccc2OCOc2c1	4.92881	0.9973
37	GAJFEF	2,6–Dibromoaniline	Nc1c(Br)cccc1Br	1.04970	0.9827
36	HBRBEN02	Hexabromobenzene	Brc1c(Br)c(Br)c(Br)c(Br)c1Br	0.00791	0.9912
35	LOBVEF	2–Bromo–5–nitropyridine	[O–][N+](=O)c1ccc(Br)nc1	1.519	0.9929
41	MOLJED	3,6–Bis(methylthio)–1,2,4,5–tetrazine	CSC1NNC(NN1)SC	0.68675	0.9853
34	SIQZEX	bis(2–Pyridyl) ketone	O=C(c1ccccn1)c2ccccn2	2.88906	0.9901
32	SULAMD07	Sulfanilamide	Nc1ccc(cc1)[S](N)(=O)=O	0.11286	0.9936
43	TBENZA02	Tribenzylamine	C(N(Cc1ccccc1)Cc2ccccc2)c3ccccc3	0.86552	0.9959
42	VOQTIF	2,9–Dichloro–1,10–phenanthroline	Clc1ccc2ccc3ccc(Cl)nc3c2n1	0.07138	0.9806
38	WEMDEX	2,6–Dichloroaniline	Nc1c(Cl)cccc1Cl	1.86728	0.9898
27	XEVRUL	2,4–Dihydroxybenzaldehyde	Oc1ccc(C=O)c(O)c1	1.57232	0.9824
39	YIFXER	2,3,4,5,6–Pentafluoroaniline	Nc1c(F)c(F)c(F)c(F)c1F	18.54291	0.9944
40	ZIZDOD	3,6–Dichloro–1,2,4,5–Tetrazine	C1(=NN=C(N=N1)Cl)Cl	0.38202	0.9992
**Test Compounds**					
A	DCDHNQ	2,3-Dichloro-5,8-dihydroxynaphtho-1,4-quinone	ClC1=C(Cl)C(=O)c2c(C1=O)c(O)ccc2O	0.01652	0.9859
B	DHXANT	1,4-Dihydroxyanthraquinone	[C]1=[C][C]=c2c(=[C]1)c(c1C(=O)[C][C][C](c1c2[O])[O])[O]	0.02154	0.9915
C	JUGLON	Juglone	C1(=C2C(=[C][C]=[C]1)C(=O)[C]=[C]C2=O)[O]	0.33738	0.9802
D	KOFHOD	Benzo(a)fluoren-11-one	O=C1c2ccccc2c3ccc4ccccc4c13	0.09534	0.9768
E	TBBENQ	Tetrabromo-p-benzoquinone	Br[C]1C(=O)[C](Br)[C](C(=O)[C]1Br)Br	0.04999	0.9858

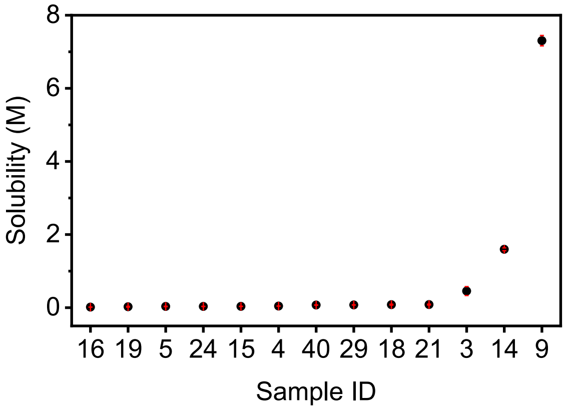

Fig. S2. Standard deviation of CV solubility measurements for selected compounds using 1:1 DOL:DME electrolyte (1 M LiTFSI and 0.2 M $LiNO_3$). The size of the error bars are typically smaller than the datapoints themselves.

Table S2. Standard measurements of triplicates and standard deviation for selected test set.

ID	Compound Name	Trial 1 (M)	Trial 2 (M)	Trial 3 (M)	Standard Deviation
17	2–((4–(1,1–Dimethylethyl)cyclohexyl)methyl)–3–hydroxy–1,4–naphthalenedione	0.01596	0.01532	0.01553	3.26241E-4
20	1,5–Dichloroanthraquinone	0.02536	0.03641	0.04844	0.01154
5	Tetrachloro–p–benzoquinone	0.03323	0.02660	0.03758	0.00553
25	Acenaphthenequinone	0.03367	0.03039	0.03295	0.00172
16	2–Methoxynaphthalene–1,4–dione	0.03574	0.03033	0.03327	0.00271
4	Chloranilic acid	0.04432	0.04295	0.04351	6.88791E-4
42	2,9–Dichloro–1,10–phenanthroline	0.07138	0.07946	0.07782	0.00427
30	2–Cyano–3–(4–hydroxyphenyl) propenoic acid	0.07490	0.07341	0.07332	8.87374E-4
19	alpha–Chloroanthraquinone	0.08263	0.10541	0.09082	0.01154
22	1–hydroxy–9,10–anthraquinone	0.08512	0.05966	0.07197	0.01273
3	2,5–Dichloro–1,4–benzoquinone	0.45473	0.69240	0.52955	0.12152

15	2–Bromo–1,4–naphthoquinone	1.59704	1.66165	1.66537	0.03842
9	2,3,5,6–Tetrafluorohydroquinone	7.30489	7.14303	7.42492	0.14146

Measurement of diffusion coefficient as a function of concentration

To validate the assumption that the diffusion coefficient is relatively consistent regardless of concentration, the diffusion constant of alpha-chloroanthraquinone was measured at seven different concentrations. For each concentration, the measurement was taken using scan rates of 25 mV/s, 50 mV/s, 75 mV/s, 100 mV/s, 125 mV/s, and 150 mV/s. The resulting peak concentration for each measurement was plotted against the square root of the scan rate (**Fig. S3**). Considering the Randles Sevcik equation (eq 2), the slope is equal to $2.69 \times 10^5 n^{\frac{3}{2}} AC\sqrt{D}$. A summary of the diffusion constants can be found in Table S3.

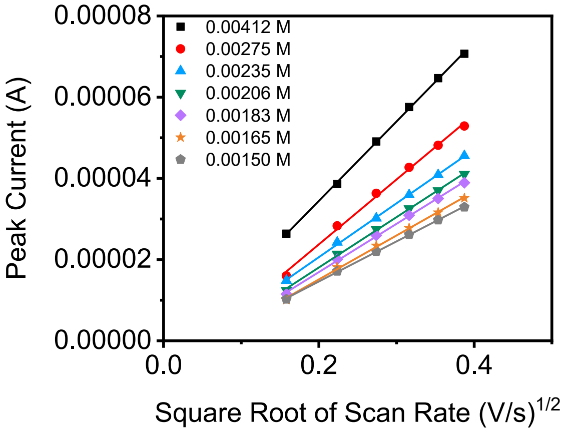

Fig. S3. Correlation of the square root of the scan rate and peak current response at different concentrations to validate consistency of the diffusion coefficient.

Table S3. Table of diffusion coefficients at different concentrations calculated from the slope of the square root of the scan rate vs peak current response.

Concentration of alpha–Chloroanthraquinone (M)	Slope	Diffusion Coefficient (cm^2s^{-1})	R^2 of Curve
0.004121	0.0002	6.5 E-06	0.9996
0.002747	0.0002	1.5 E-05	0.9966
0.002354	0.0002	2.0 E-05	0.9994
0.002061	0.0001	6.5 E-06	0.9991
0.001832	0.0001	8.2 E-06	0.999
0.001649	0.0001	1.0 E-05	0.9984
0.001499	0.0001	1.2 E-05	0.9996

Variable time point study of solubility measurements with CV methods

To determine if the measured solubility is at equilibrium, the solubility was measured after forming a saturated solution immediately, after 1 hour, after 2 hours, and after 24 hours. Saturated solutions of redox–active compounds were prepared by stirring the analyte (20–50 mg) in 1:1 DOL:DME electrolyte (1 M LiTFSI and 0.2 M $LiNO_3$, 0.5 mL) for each time length. At each time point, a portion of the saturated solution was filtered and an aliquot was diluted for a CV measurement. Despite sealing the saturated solution between time points, some solvent evaporated leading to higher concentrations of salt in the later time measurements. This inadvertently led to lower solubilities measured for the later points as seen in Fig. S4 and Table S4.

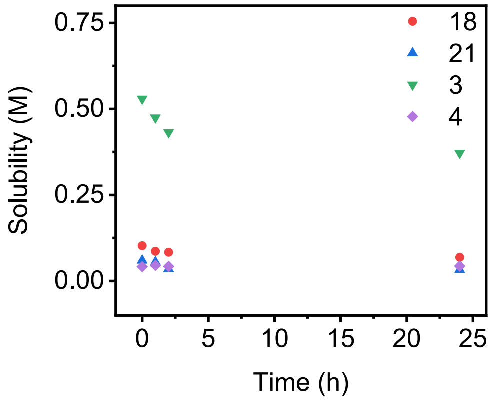

Fig. S4. Solubility of selected compounds measured at various points over a 24 hour time period using CV.

Table S4. Solubility measurements of selected compounds at time points immediately, 1 hour, 2 hours, and 24 hours.

ID	Compound Name	Immediate (M)	1 hour (M)	2 hours (M)	24 hours (M)
4	Chloranilic acid	0.04163	0.04540	0.04245	0.04347
22	1–hydroxy–9,10–anthraquinone	0.05966	0.05513	0.03507	0.03264
19	alpha–Chloroanthraquinone	0.10260	0.08634	0.08365	0.06886
3	2,5–Dichloro–1,4–benzoquinone	0.52955	0.47491	0.43214	0.37201

Determination of the solubility of redox–active compounds in pure 1:1 DOL:DME

Saturated solutions of redox–active compounds were prepared by sonicating the analyte (20–50 mg) in pure 1:1 DOL:DME solvent without added salt for 10 min. If the mixture became homogeneous, additional analyte was added until saturation was reached. The resulting suspension was then filtered through a glass fiber plug, and the saturated solution was diluted with 1:1 DOL:DME electrolyte (1 M LiTFSI and 0.2 M $LiNO_3$) for CV analysis. The solubility of the analyte was determined using the calibration curves described above.

Table S5. Solubilities of anthraquinones in 1:1 DOL:DME electrolyte (1 M LiTFSI and 0.2 M $LiNO_3$) and pure 1:1 DOL:DME (without salts).

**Compound Name**	**Solubility in electrolyte (M)**	**Solubility in pure solvent (M)**	**R^2 of Calibration Curve**
1–Aminoanthraquinone	0.05117	0.03908	0.9709
1–Amino–2–methylanthraquinone	0.04322	0.07991	0.9873
1–Amino–4–hydroxyanthraquinone	0.04673	0.12081	0.997
1–Amino–5–chloroanthraquinone	0.17276	0.60821	0.9934
2–Aminoanthraquinone	0.01238	0.01562	0.9862
Anthraquinone 2–carboxylic acid	0.00299	0.02788	0.9844
1,2–Diaminoanthraquinone	0.00633	0.00657	0.9713
1,5–Diaminoanthraquinone	0.00890	0.00623	0.9847
1,4–Dichloroanthraquinone	0.07284	0.08626	0.992
2,3–Dimethylanthraquinone	0.02603	0.02272	0.9918
2–Methylanthraquinone	0.13976	0.36027	0.9948

Comparison of solubility measurements with UV–vis and CV methods

To evaluate the accuracy of the CV method for OEMs compared to the UV–vis method, a set of ten anthraquinone solubilities was measured using the CV method in 1:1 DOL:DME electrolyte (1 M LiTFSI and 0.2 M $LiNO_3$) and compared to the solubilities measured by UV–vis in Tuttle et. al. (**Fig. S5**).[1]

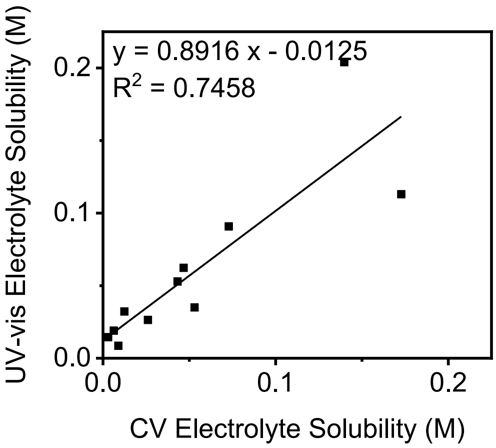

Fig. S5. Correlation between solubilities of anthraquinones in 1:1 DOL:DME electrolyte (1 M LiTFSI and 0.2 M $LiNO_3$) using the UV–vis and CV methods.

Although differences were observed between the solubilities measured by the two methods, we believe that the CV-based approach provides more reliable values. Several quinone samples examined in this study, such as 1-

amino-5-chloroanthraquinone, exhibit intense UV–vis absorption that extends beyond the linear regime of Beer's law, leading to inaccuracies in solubility analysis. Notably, most of the discrepancies between the two methods occur in the high-solubility regime (>0.1 M). In contrast, in the low-solubility range (<0.1 M, which is most relevant for the design of solid-state OEMs, the solubilities obtained from both methods are in close agreement.

Dataset Construction for Lattice Energy Calculations

Quinones represent the most common and widely studied class of organic electrode materials. Their structural diversity and rich electrochemical behavior make them an excellent starting point for probing the correlation between solubility and molecular structure. To build a representative dataset, we used the Cambridge Structural Database (CSD) accessed through the Cambridge Crystallographic Data Centre (CCDC)[2,3] and generated a collection of quinone crystal structures using ConQuest. To simplify subsequent lattice energy calculations, only single–component XRD structures of quinones—those without co–crystallized solvents or counterions—were selected. Based on this criterion, a set of unique structures were compiled for this study.

Lattice Energy Calculation using Periodic DFT

Selection of K-point mesh and expansion size for lattice energy calculations

Utilizing the crystal structure NEFJUG (2,5-dihydroxy-3,6-dimethoxy-benzoquinone), geometry optimizations were done with varying k-points to generate a Monkhorst-Pack grid.[4] As k-points vary based on the size of the unit cell, the k-points were selected by taking a constant, N, dividing by the lattice parameters, and rounding to the nearest whole number. No shift was applied to the k-point grid. The results of these variations are seen in Table S6 and Fig. S6. Additional calculations were performed using varying expansion sizes for the isolated molecule calculations. The lattice parameters of the unit cell were multiplied by various expansion factors to determine the point at which the molecule experienced no interactions with the next closest molecule. The results of these calculations are seen in Table S7 and Fig. S7.

Table S6. Full unit cell calculation energies based on varying k-points.

Constant, N	K points	Total Energy (Ryd)	Computation Time
None	Gamma point	-406.9000373036	6 minutes 11 seconds
10	2, 2, 1	-406.8344631085	20 minutes 34 seconds
15	3, 3, 2	-406.8358806473	47 minutes 12 seconds

20	4, 4, 3	-406.8358095225	1 hour 40 minutes
25	5, 5, 4	-406.8358089182	3 hours 21 minutes
30	6, 5, 4	-406.8358318611	4 hours 20 minutes
35	7, 6, 5	-406.8358093189	6 hours 41 minutes
40	8, 7, 6	-406.8358092950	10 hours 12 minutes
45 (Very Fine)	9, 8, 6	-406.8358092409	13 hours 46 minutes

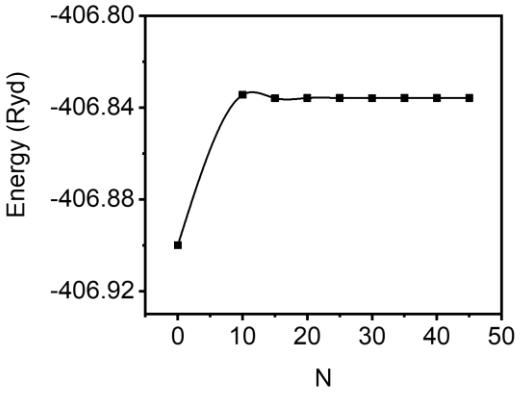

Fig. S6. Plot of change in energy based on increasing N or “fineness” of k-point mesh.

Table S7. Isolated molecule calculation energies based on varying expansion factors.

Expansion Size	Total Energy (Ryd)	Computation Time
None	-406.8358092409	13 hours 46 minutes
Doubled	-406.9042105005	53 minutes 12 seconds
Tripled	-406.7233044130	48 minutes 34 seconds
Quadrupled	-406.7265447643	1 hour 37 minutes
Quintupled	-406.7266497081	3 hours 59 minutes
Sextupled	-406.7266047213	5 hours 54 minutes

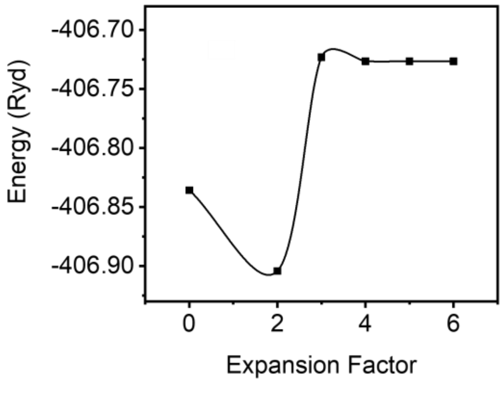

Fig. S7. Plot of change in energy based on increasing expansion factors.

Method of lattice energy calculations for quinone with known crystal structures

A set of quinones with both known crystal structures and reported sublimation enthalpies in the National Institute of Standards and Technology (NIST) database was used to determine the accuracy of the method of lattice energy calculations.[5,6] Initial single-point self-consistent field (SCF) energy calculations were performed using the reported crystal structures from the Cambridge Structural Database (CSD), yielding $E_{cryst,SCF}$. A corresponding SCF calculation was conducted for an isolated molecule extracted from the crystal structure, denoted as $E_{mol,SCF}$. Structural relaxations were then carried out for both the full unit cell ($E_{cryst,Relax}$) and the isolated molecule ($E_{mol,Relax}$).

Using these energies, lattice energies were calculated by subtracting the summed isolated-molecule energies from the unit-cell energy using four different combinations: $E_{cryst,SCF} - \Sigma E_{mol,SCF}$, $E_{cryst,SCF} - \Sigma E_{mol,Relax}$, $E_{cryst,Relax} - \Sigma E_{mol,SCF}$, and $E_{cryst,Relax} - \Sigma E_{mol,Relax}$. In comparing the calculated lattice energies using each method to experimental data, it was determined that $E_{cryst,Relax} - \Sigma E_{mol,Relax}$ exhibits the strongest correlation with the experimental lattice energy from NIST (Fig. 4).

Table S8. Calculated and experimental sublimation enthalpies of crystal structures.

Crystal Name	Compound Name	NIST Lattice Energy (kJ/mol)	SCF Full Unit Cell (Ryd)	Relaxed Full Unit Cell (Ryd)	SCF Isolated Molecule (Ryd)	Relaxed Isolated Molecule (Ryd)
ANTQUO	Anthraquinone	–132.603	–701.097	–701.806	–349.467	–350.817
BNZQUI	Benzoquinone	–67.273	–397.114	–397.149	–198.447	–198.52
CLBENQ	2–Chloro–benzoquinone	–73.994	–1106.83	–1107	–276.652	–276.692
DHANQU01	1,8–Dihydroxyanthraquinone	–114.958	–1735.34	——	–433.757	–434.174
DHANTQ02	1,5–Dihydroxyanthraquinone	–122.558	–868.533	–868.533	–433.764	–434.18
DHXANT	1,4–Dihydroxyanthraquinone	–129.058	–1693.24	–1737.03	——	–434.178
DOGWOL	2,5–Dimethylbenzoquinone	–105.758	–970.069	–971.004	–242.422	–242.657
EQAHEL	1,4–Diaminoanthraquinone	–109.085	——	–3278.18	–409.29	–409.698
HESKOF	2,5–bis(1,1–dimethylethyl)–benzoquinone	–127.358	–2201.41	–2206.07	–364.406	–367.581
JUNBIF	1–Hydroxyanthraquinone	–126.553	–1569.19	–1570.29	–392.097	–392.498
NAPHQU	Naphthoquinone	–95.958	–1066.36	–1098.96	–266.44	–274.669
NEQZEQ	1,5–Dipiperidylanthraquinone	–180.417	–1224.72	–1226.3	–611.979	–613.019
OGAGAI	Hydroquinone	–106.121	–1806.87	–1809.73	–199.292	–200.995
PENTQU	6,13–Pentacenedione	–120.958	–1005.55	–1006.33	–502.55	–503.044
SNQUOX	2–Nitroso–1–naphthol	–61.458	–1214.19	–1216.29	–303.27	–304.002

TCBENQ	Tetrachloro–p–benzoquinone	–103.699	–1022.48	–1022.51	–511.018	–511.192

Details of lattice energy calculation

Initial input files were generated using an online QE input generator and Avogadro.[7,8] Subsequent input files were generated using a custom Python script with an ase parser to read the cif files.[9] All cif files were visualized with Mercury, while relaxed output geometries were visualized with Avogadro.[10]

Table S9. Calculated lattice energies of crystal structures.

ID	Crystal Name	Full Lattice Relaxed Energy (Ryd)	Isolated Molecule Relaxed Energy (Ryd)	Lattice Energy (Ryd)	Lattice Energy (kJ/mol)
**Quinones**					
25	ACNAQU	–1246.478663	–311.5296401	–0.090025704	–118.181264
18	ANTQUO	–701.8058149	–350.8165898	–0.086317688	–132.603512
1	BNZQUI	–397.1486049	–198.5200549	–0.054247506	–71.21168
24	BOSMUS	–819.6320933	–409.7024711	–0.113575507	–149.092656
21	BRANTO	–1108.135883	–553.9764302	–0.091511385	–120.131008
15	BRNAPQ	–1911.697225	–477.8480746	–0.076231746	–100.072912
3	BZQDCL10	–709.8521985	–354.8636541	–0.062445126	–81.972928
20	CANTRQ01	–1014.43462	–507.1251687	–0.092141324	–120.955256
4	CLANAC	–876.5826602	–438.2126637	–0.07866638	–103.269488
19	CLANTO	–858.1110702	–428.9710332	–0.084501875	–110.926208
2	CLBENQ	–1106.995414	–276.6921929	–0.056660664	–74.378968
23	DHANTQ01	–868.5330414	–434.1795983	–0.086922349	–122.557728
7	DHXBZQ	–1127.852215	–281.8892753	–0.073778423	–96.851232
9	GUFMAV	–867.4516268	–433.6474294	–0.078383998	–102.897112
12	GUFMEZ	–2027.568746	–1013.696665	–0.087708267	–115.135312
13	HIQTEG	–1460.775321	–365.1102684	–0.083561925	–109.696112
10	IBOVAZ	–632.8388836	–316.2836056	–0.135836133	–178.317896
11	JOYQUJ01	–1214.890301	–607.3442637	–0.100886514	–132.3631064
22	JUNBIF	–1570.287261	–392.4976777	–0.074137566	–97.324024
14	NAPHQU	–1098.957151	–274.6692772	–0.070010654	–91.905744
26	SNQUOX	–1216.294111	–304.0023105	–0.071217389	–93.487296
5	TCBENQ	–1022.507862	–511.191628	–0.062303096	–81.788832
6	TCLOBQ	–1022.45064	–511.1637747	–0.061545371	–80.79304
8	TFBENQ	–862.4682104	–431.176474	–0.057631206	–75.655088
17	UHUKAJ	–1086.302428	–543.0367194	–0.114494343	–150.301832
16	UPAGUO	–1348.835181	–337.1208348	–0.087960401	–115.470032
**Non–Quinones**					

29	AMPHOL01	–755.4312682	–188.7645008	–0.09331622	–122.499152
33	AZOBEN01	–1157.710667	–289.3545254	–0.07314129	–95.96137248
44	CORONE	–913.6941057	–456.7426273	–0.104425537	–137.084576
31	DUNHIE	–1328.827	–332.1205665	–0.086183625	–113.13536
30	FEVBIS	–1383.430548	–345.7281958	–0.129441062	–169.920608
28	FOMZUD	–1118.012946	–279.4326238	–0.070612631	–92.69652
37	GAJFEF	–2214.233356	–553.489771	–0.068567965	–90.010392
36	HBRBEN02	–2673.605513	–1336.714553	–0.088203746	–115.788016
35	LOBVEF	–879.2920452	–439.57895	–0.06707261	–88.048096
41	MOLJED	–1304.478173	–326.052483	–0.067060238	–88.03136
34	SIQZEX	–1241.253479	–310.2271994	–0.086170383	–113.118624
32	SULAMD07	–1304.773586	–326.0833294	–0.110067167	–144.490256
43	TBENZA02	–1761.223949	–440.206162	–0.099825238	–131.04288
42	VOQTIF	–1774.087225	–443.4304966	–0.091309677	–119.863232
38	WEMDEX	–1214.132443	–303.4773608	–0.055749863	–73.186528
27	XEVRUL	–1044.714724	–261.0947712	–0.083909725	–110.152168
39	YIFXER	–1752.018567	–437.9458636	–0.058778171	–77.161328
40	ZIZDOD	–1232.923892	–308.1814819	–0.049491124	–64.969152
**Test Compounds**					
A	DCDHNQ	–2057.753554	–514.3691268	–0.069261694	–90.87134253
B	DHXANT	–1737.02673	–434.1782	–0.0784825	–102.96904
C	JUGLON	–1265.694232	–316.35012	–0.073437923	–96.35055498
D	KOFHOD	–1467.729058	–366.8445473	–0.087717212	–115.0849821
E	TBBENQ	–2022.5802	–1011.215269	–0.074830967	–98.1782287

Calculation of sublimation enthalpy from lattice energy

ΔH_{sub} was calculated from U_{latt} using equation S1.[11,12]

$$\Delta H_{sub} = -U_{latt} - 2RT \quad \text{(eq S1)}$$

The temperature was set to 298K for all calculations.

DFT Computational Details

Sublimation entropy computations

Sublimation free energy was calculated using the previously calculated ΔH_{sub} as well as sublimation entropy (ΔS_{sub}). ΔS_{sub} consists of 3 terms as seen in equation S2: vibrational entropy, rotational entropy, and translational entropy (S_{vib}, S_{rot}, and S_{trans} respectively).

$$\Delta S_{sub} = S_{vib} - (S_{trans} + S_{rot}) \quad \text{(eq S2)}$$

S_{rot} and S_{trans} are related to the gas phase of a molecule, while S_{vib} is related to the phonon states of the crystal

phase of a molecule. Using a constant term of 513.65 J mol^{-1}K^{-1} in place of S_{vib}, ΔG_{sub} was calculated for this same dataset. This was validated using the experimental terms from McDonagh et. al., and the use of a constant value for S_{vib} had a relatively minor effect on the overall sublimation energy prediction.[12]

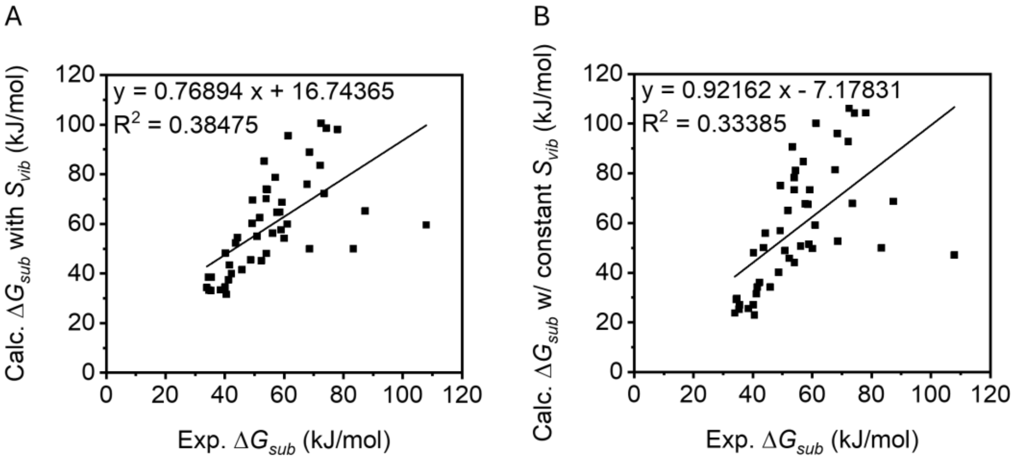

Fig. S8. Comparison of calculated ΔG_{sub} terms (A) with S_{vib} using ΔH_{sub} and ΔS_{sub} from McDonagh et al. and (B) without S_{vib} using S_{trans} and S_{rot} from DFT geometry optimizations.

Solvation energy computations

The properties of 1:1 mixture of DOL:DME were defined using the average values of DOL and DME as follows:

Eps=7.445

EpsInf=1.92442788

HbondAcidity=0.

HbondBasicity=0.65

SurfaceTensionAtInterface=41.1358326

CarbonAromaticity=0.

ElectronegativeHalogenicity=0.

This calculation used the same DFT method as the sublimation entropy calculations.

Table S10. Calculated ΔH_{sub}, ΔS_{sub}, and ΔG_{solv} of redox active molecules.

ID	Crystal Name	ΔG_{solv} (kJ/mol)	ΔH_{sub} (kJ/mol)	ΔS_{sub} (J/molK)
**Quinones**				

25	ACNAQU	–49.7768545	113.2248768	303.804424
18	ANTQUO	–47.826108	108.1958882	309.264544
1	BNZQUI	–34.4229305	66.25797216	283.40324
24	BOSMUS	–49.3882805	144.1408053	314.276976
21	BRANTO	–51.979649	115.1743105	319.553
15	BRNAPQ	–43.1395905	95.12533612	311.444408
3	BZQDCL10	–38.789137	77.01939768	301.72916
20	CANTRQ01	–52.515251	116.001343	319.624128
4	CLANAC	–41.2387285	98.31384828	307.50308
19	CLANTO	–50.283576	105.9784968	314.841816
2	CLBENQ	–37.261096	69.42584503	293.838136
23	DHANTQ01	–66.388393	109.1518721	314.473624
7	DHXBZQ	–56.9917285	91.8971824	293.536888
9	GUFMAV	–20.295115	97.94315111	302.227056
12	GUFMEZ	–40.7451345	110.1835856	329.247328
13	HIQTEG	–42.590861	104.7404755	312.615928
10	IBOVAZ	–47.216992	173.3634417	300.499064
11	JOYQUJ01	–46.1956725	127.4833289	313.406704
22	JUNBIF	–57.7898805	92.36875988	311.971592
14	NAPHQU	–39.3903765	86.94690362	298.670656
26	SNQUOX	–40.8081465	88.53518488	302.268896
5	TCBENQ	–37.880714	76.83294695	313.159848
6	TCLOBQ	–35.449501	75.83824332	312.917176
8	TFBENQ	–25.498856	70.69992322	301.846312
17	UHUKAJ	–53.1900045	145.347007	329.184568
16	UPAGUO	–44.665006	110.5145744	305.637016
**Non–Quinones**				
29	AMPHOL01	–36.8961515	117.5454256	284.043392
33	AZOBEN01	–30.5529435	91.06078641	306.298088
44	CORONE	–51.533314	132.129182	322.624056
31	DUNHIE	–39.1593325	108.1821115	308.7792
30	FEVBIS	–54.458121	164.9683125	307.946584
28	FOMZUD	–29.7757955	87.74128902	296.398744
37	GAJFEF	–31.4771195	85.05715425	310.297992
36	HBRBEN02	–38.8810295	110.8340256	337.054672
35	LOBVEF	–31.4088565	83.0941273	303.662168
41	MOLJED	–49.6718345	83.07788514	302.193584
34	SIQZEX	–43.052949	108.1647288	305.34832
32	SULAMD07	–57.934283	139.5352313	300.499064
43	TBENZA02	–51.170995	126.0901387	324.90852
42	VOQTIF	–51.4256685	114.9113364	317.201592
38	WEMDEX	–30.051473	68.23019065	298.13092
27	XEVRUL	–38.2089015	105.1970489	293.197984

39	YIFXER	–38.920412	72.20560172	302.423704
40	ZIZDOD	–17.254786	60.01403156	292.75448
**Test Compounds**				
A	DCDHNQ	-72.153991	85.9678468	316.235088
B	DHXANT	-69.712276	98.07245988	314.548936
C	JUGLON	-51.3626565	91.45019201	301.963464
D	KOFHOD	-48.9209415	110.1953278	314.289528
E	TBBENQ	-43.89836	93.27891026	329.243144

Calculation of Additional Solubility Descriptors

For the dataset, the molar volume (V_m) of each molecule was calculated using RDKit Chem.[13] The terms AlogP and bRot were also calculated using the RDKit package, quantitative estimation of drug–likeness (QED).

Table S11. Calculated V_m, AlogP, and bRot of redox active molecules.

ID	Crystal Name	V_m (L/mol)	AlogP	bRot
**Quinones**				
25	ACNAQU	0.086384386	1.01994	0
18	ANTQUO	0.109667846	2.462	0
1	BNZQUI	0.05757032	0.2506	0
24	BOSMUS	0.123426912	1.6264	0
21	BRANTO	0.114875672	1.86403	0
15	BRNAPQ	0.090060214	1.34835	0
3	BZQDCL10	0.076310784	1.3836	0
20	CANTRQ01	0.119298229	2.56994	0
4	CLANAC	0.086943227	1.155	0
19	CLANTO	0.109966538	1.71673	0
2	CLBENQ	0.066988728	0.8171	0
23	DHANTQ01	0.117221843	2.7496	0
7	DHXBZQ	0.068520725	0.022	0
9	GUFMAV	0.073555117	1.6542	0
12	GUFMEZ	0.107610731	4.1478	0
13	HIQTEG	0.136053842	3.0832	0
10	IBOVAZ	0.086750523	–2.6038	0
11	JOYQUJ01	0.084389899	2.6228	0
22	JUNBIF	0.114220478	2.1676	0
14	NAPHQU	0.076729915	0.42894	0
26	SNQUOX	0.09091293	1.4608	0
5	TCBENQ	0.094588758	2.5166	0
6	TCLOBQ	0.094010646	2.5166	0

8	TFBENQ	0.07081872	1.4394	0
17	UHUKAJ	0.191027475	5.1203	2
16	UPAGUO	0.099030586	1.5959	1
**Non–Quinones**				
29	AMPHOL01	0.062797416	0.9744	0
33	AZOBEN01	0.104074613	4.102	2
44	CORONE	0.155526581	6.9192	0
31	DUNHIE	0.115265898	1.9293	3
30	FEVBIS	0.100071187	1.38378	2
28	FOMZUD	0.076768456	1.2278	1
37	GAJFEF	0.080892322	2.7938	0
36	HBRBEN02	0.121273445	6.2616	0
35	LOBVEF	0.07501485	1.7523	1
41	MOLJED	0.093658962	–0.5184	2
34	SIQZEX	0.099680962	1.7076	2
32	SULAMD07	0.0861146	–0.0838	1
43	TBENZA02	0.171540283	4.889	6
42	VOQTIF	0.115694664	4.0898	0
38	WEMDEX	0.076122898	2.5756	0
27	XEVRUL	0.07216283	0.9103	1
39	YIFXER	0.073584022	1.9643	0
40	ZIZDOD	0.059535901	0.5734	0
**Test Compounds**				
A	DCDHNQ	0.112095917	2.166	0
B	DHXANT	0.11138773	1.99763	0
C	JUGLON	0.08151861	0.77255	0
D	KOFHOD	0.123995389	4.0512	0
E	TBBENQ	0.106998896	2.84576	0

Data–Driven Solubility Modeling

Linear regression models derived from the thermodynamic equation of solution

To establish baseline relationships between molecular descriptors and target property (logS), we evaluated two analytically motivated linear models. Both models were constructed using standard linear models using the least squares regression approach.

Model–1 (Fig. 5B) was derived from the thermodynamic equation for solubility:

$$\mathrm{logS} = 0.03382946 \times \Delta G_{solv} - 0.00486614 \times (\Delta H_{sub} - \Delta S_{sub}\mathrm{T}) - 0.93424683 \times \log(V_m) -$$

0.001545181802739104 (eq S3)

Model–2 extends Model–1 by incorporating additional flexibility terms (AlogP and bRot), which have been shown to be good predictors of solubility. These were intended to account for uncertainty in ΔS_{sub}.

$$\text{logS} \approx 0.350126 \times \Delta G_{solv} - 0.172557 \times (\Delta H_{sub} - \Delta S_{sub}\text{T}) - 0.078295 \times \log(V_m) - 0.082808 \times \text{AlogP} + 0.235689 \times \text{bRot} - 0.5400235917 \quad \text{(eq S4)}$$

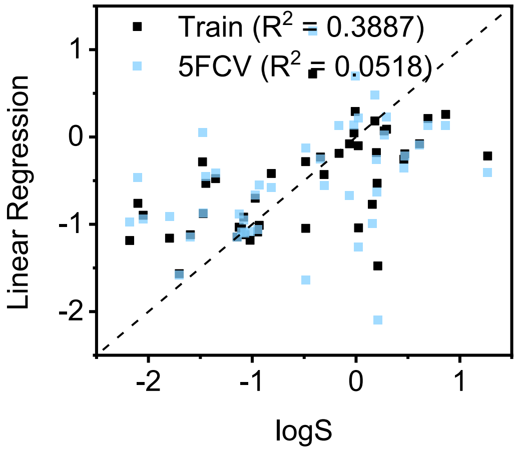

Fig. S9. Linear regression model (eq S4) including additional descriptors AlogP and bRot.

For each model, the coefficients were estimated using a form of linear regression via scikit–learns "LinearRegression" module.

Symbolic regression model with SyMANTIC

Beyond linear baselines, we applied SyMANTIC – a symbolic regression framework designed to recover interpretable analytical expressions from numerical data. SyMANTIC searches over symbolic expressions composed of elementary mathematical operators {+, –, exp, ×, etc.} with the input feature space. This method optimizes a multi–objective Pareto front between prediction accuracy and symbolic complexity of the analytical expression, yielding a set of models that trade interpretability and accuracy.

The preprocessed feature space, along with the target (logS), is provided as an input parameter, along with algorithm hyperparameters like stopping criteria (when does SyMANTIC stops searching the space (R^2 and RMSE meeting criteria) and the feature screening conditions to avoid computational blowout – mutual information with top 10% features that are showing relational dependency with the logS)

```
symantic = SymanticModel(
    df,
    operators=['+', '–', '*', '/'],
    metrics=[0.05, 0.99],
    initial_screening=[mi, 0.90],
)
res, pareto = symantic.fit()
```

SyMANTIC produces a set of Pareto–optimal expressions, from which the final expression balancing complexity and maintaining near-optimal loss is selected (Eq. 6).

To assess the robustness of the fixed model structure, we have opted to re-optimize the coefficients using a 5-fold cross-validation procedure. This tests the stability of the discovered equation relationship, not the stability of the symbolic search procedure. The performance of the symbolic model is quantified using Train R^2 and 5–fold CV $R^{2,}$ which sets a direct comparison with the linear baselines.

Random Forest regression model baseline

To benchmark the SyMANTIC analytical model, we opted for the widely used Random Forest combined with the feature selection procedures that are kept symmetric, as in the SyMANTIC framework. We employed the RandomForestRegressor from the scikit–learn package with the hyperparameters optimized using grid–search.

```
from sklearn.ensemble import RandomForestRegressor
from sklearn.feature_selection import SelectKBest, mutual_info_regression, VarianceThreshold

X = df.drop(columns=['Target'])
y = df['Target'].values

# Keep top 10% features by mutual information
k_keep = max(1, int(np.ceil(0.10 * X.shape[1])))

def mi_score(X_arr, y_arr):
    return mutual_info_regression(X_arr, y_arr, random_state=42, n_neighbors=5)

rf = RandomForestRegressor(
```

n_estimators=500,

random_state=42,

n_jobs=–1,

)

Determination of additional equations to model solubility

Outside of linear regression, additional models, besides those in Figure 5, were considered using the same solubility descriptors. The following equations were found and plotted.

$$\log S = 0.471434 \times ((V_m + \text{RotB}) + (V_m \times \Delta G_{solv})) - 0.5400235917 \quad \text{(eq S5)}$$

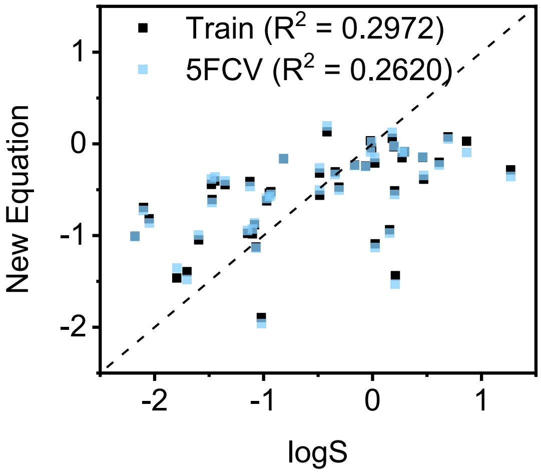

Fig. S10. Solubility model constructed without considering the thermodynamic solubility equation.

Interpretability of SyMANTIC Model

$$logS = 1.551 + \frac{0.30 \times \Delta G_{solv}\,(Xp_{4dv} - Xp_{6dv})}{\text{ETA}_{\alpha}} - \frac{0.0047 \times Xp_{7dv}\,(\Delta H_{sub} - \text{ASA})}{JG12} - \frac{0.0402 \times MWC07}{Xp_{6dv} \times (\text{ETA}_{\alpha} - \text{ATSC3s})} \quad \text{(eq S6)}$$

Xp_{4dv}*, *Xp_{6dv}*, and *Xp_{7dv} are 2D **Kier–Hall valence molecular connectivity (χ) path indices** of orders 4, 6, and 7, respectively, weighted by the atomic property dv (valence electrons). In Mordred, χ descriptors are computed by enumerating the relevant subgraphs and summing contributions of the form $(\prod_i P_i)^{-1/2}$, where P_i is the chosen atomic property for each atom in the path; for dv, Mordred defines this property as the number of valence electrons. These descriptors therefore encode how valence-electron-weighted connectivity is distributed over paths of increasing topological length. This is consistent with the original valence χ framework of Kier and Hall, which ties the valence connectivity index to the electronic and volumetric characteristics of bonded atoms.[14,15]

ETA_{α} is the **ETA core count** from the Extended Topochemical Atom (ETA) descriptor family. Mordred defines it as

$$\propto = \sum_{i=1}^{A} \frac{Z_i - Z_i^v}{Z_i^v} \times \frac{1}{PN_i - 1} \qquad \text{(eq S7)}$$

where Z_i is the total number of electrons, Z_i^v is the number of valence electrons, and PN_i is the periodic number. In practice, ETA_α is a graph-based measure of molecular core constitution that reflects atom types and molecular framework size in a chemically weighted way, rather than simple atom count alone.[16,17]

JGI2 is the **2nd-order mean topological charge index**. In the Mordred implementation, the underlying charge-term matrix is constructed from the adjacency matrix and inverse-squared topological distances, and JGI2 is obtained as the mean absolute charge-transfer term for atom pairs separated by a graph distance of 2. Thus, JGI2 is best interpreted as a 2-bond electronic/topological charge-distribution descriptor.[17]

ATSC3s is the **centered Moreau–Broto autocorrelation of lag 3**, weighted by the atomic properties, which Mordred defines as **an intrinsic state**. In the Mordred formulation, ATSC descriptors are computed using centered atomic property values, so ATSC3s capture how intrinsic-state values are correlated between atom pairs separated by three bonds. Chemically, this is a topological measure of how local electronic-state character is distributed across the molecular graph at an intermediate range.[18]

MWC07 is the **walk count descriptor of length 7**. In the Mordred descriptor list, it is defined as a "walk count (leg-7)," and in the source implementation the non-self-returning walk count for order >1 is computed from the order-7 adjacency matrix as $log(\sum A^7+1)$. It therefore reflects long-range graph connectivity / branching complexity over seven bond steps.[19]

ASA is **Labute's Approximate Surface Area**, exposed in Mordred as an RDKit wrapper. This descriptor belongs to the surface-area-based descriptor family introduced by Labute, where descriptors are built from atomic contributions to van der Waals surface area and related surface partitions. In a QSPR context, ASA is commonly interpreted as a proxy for overall molecular surface exposure / accessible size.[20]

The symbolic-regression model indicates that solubility is governed by a coupled balance between **(i) valence-electron-weighted molecular topology**, **(ii) surface exposure**, and **(iii) external thermodynamic stabilization / destabilization terms**. The χ-path descriptors (Xp_{4dv}, Xp_{6dv}, Xp_{7dv}) encode how valence-electron-weighted connectivity is distributed over progressively longer topological paths, while MWC07 captures longer-range graph complexity. ETA_α provides a chemically weighted measure of the molecular core framework; JGI2 quantifies short-range topological charge distribution, and ATSC3s measures intermediate-range intrinsic-state autocorrelation. Within this model, solubility is favored when topological patterns associated with more favorable medium-range valence connectivity and solvent stabilization outweigh penalties associated with greater long-range structural complexity and solid-state stabilization. Because this is a symbolic-regression expression, these terms should be interpreted as **structure–property couplings** rather than as direct thermodynamic identities.

Terms such as ($\Delta H_{sub} - ASA$) combine quantities with different physical meanings (and often different numerical scales), so that subtraction should be interpreted as a **statistical latent feature constructed by SR**, not as a literal thermodynamic difference.

10-Fold Cross-Validation and Descriptor Sensitivity Analysis of end-to-end SyMANTIC workflow

To complement the fixed-structure coefficient-refitting analysis reported in the main text, we performed an additional repeated 10-fold cross-validation of the full SyMANTIC workflow on the revised dataset of N = 44 compounds. In each fold, all model-development steps – including descriptor screening, symbolic expansion, candidate filtering, and sparse model fitting – were carried out using only the outer-training data, and performance was evaluated exclusively on the held-out fold. We included this analysis because the model – validation literature has shown that when feature selection or model tuning is performed on the same data later used for evaluation, the resulting estimate can be optimistically biased; rerunning the full discovery procedure within the training folds provides a more appropriate internal assessment of end-to-end predictive performance. We intentionally selected 10-fold rather than 5-fold cross-validation to balance the competing demands of a limited dataset (N = 44). In a standard 5-fold CV, the training partitions would be reduced to approximately 35 samples. For an end-to-end pipeline that simultaneously performs combinatorial descriptor screening and non-linear structural search, withholding 20% of the data in each split severely destabilizes the algorithm and artificially degrades training convergence. By utilizing 10-fold CV, we retain approximately 40 samples (90%) in each training split, maximizing the information available for model discovery and ensuring the structural search is driven by robust chemical trends rather than sampling artifacts. We explicitly acknowledge the statistical trade-off of this choice: 10-fold CV leaves only 4 to 5 samples in each test fold, rendering fold-wise performance metrics mathematically volatile. To address this volatility, we repeated the 10-fold partitioning across 5 random seeds (yielding 50 total outer-fold refits) and calculated our final performance metrics strictly on the aggregated, out-of-fold pooled predictions across all iterations, rather than averaging unstable fold-level scores. Figure S11 summarizes the held-out predictions by plotting the mean predicted value for each sample over all its outer-test appearances. Under this end-to-end validation protocol, the model achieved a per-sample mean R^2=0.53, RMSE = 0.35, and MAE = 0.48. These values are lower than those obtained from the fixed-structure post hoc cross-validation in the main text, which is expected because the nested analysis evaluates uncertainty in the entire symbolic discovery pipeline, whereas the fixed-structure analysis asks a narrower question: whether the final selected analytical form retains predictive utility when only its coefficients are refit on reduced data subsets. Accordingly, we present the nested 10-fold results as the more conservative internal estimate of pipeline-level predictive performance, and the fixed-structure analysis as a complementary stress test of the finalized equation.

We also examined descriptor sensitivity across the 50 refits by counting how often each descriptor appeared in the selected symbolic models (Figure S12). We interpret these frequencies as a resampling-based stability summary, rather than as evidence that any single descriptor is uniquely required in every fold. This is consistent with the variable-selection literature[35], which treats predictive accuracy and selection stability as related but distinct questions, and uses inclusion frequencies across resamples as one practical way to summarize post-selection stability.

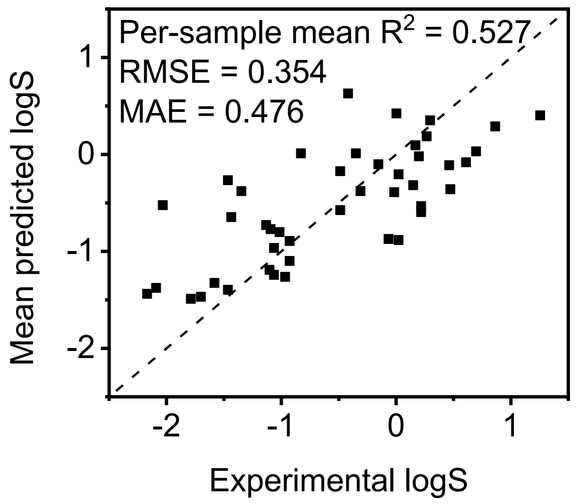

Fig. S11. Repeated nested 10-fold cross-validation parity plot for the full SyMANTIC workflow. Points show the mean held-out prediction for each sample across 5 random seeds x 10 folds.

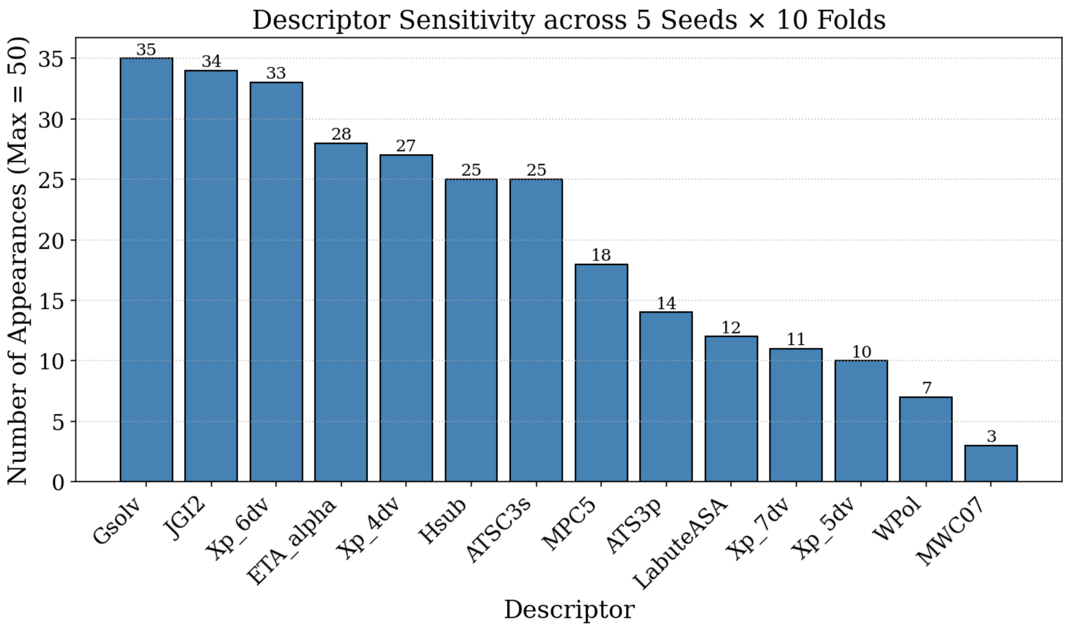

Fig. S12. Descriptor sensitivity across repeated nested 10-fold cross-validation of the full SyMANTIC workflow. Bars show how often each descriptor appeared in the selected symbolic model across 5 seeds x 10 folds.

Importantly, the repeated analysis recovers the same descriptor of families that define the final symbolic equation. In Figure S12, the most frequently selected descriptors include ΔG_{solv}, JGI2, Xp_{6dv}, ETA_{α}, Xp_{4dv}, ΔH_{sub}, and ATSC3s, all of which are primitive ingredients of Eq. 6. Thus, the dominant thermodynamic, topological, and electronic components of the final equation recur repeatedly when the full SyMANTIC workflow is rerun

under strict train/test separation. By contrast, ASA, Xp_{7dv}, and especially MWC07 appear less frequently across refits. We therefore do not interpret Eq. 6 as a unique mechanistic law or as the "only" valid symbolic representation of the data. Rather, the nested analysis supports a more cautious conclusion: Eq. S6 is one sparse representative from a broader family of related models built around a common thermodynamic–topological–electronic signal, with some terms belonging to a more stable recurrent core than others. This connection between Figure S12 and Eq. 6 is important. The repeated recovery of ΔG_{solv}, JGI2, Xp_{6dv}, ETA_{α}, Xp_{4dv}, ΔH_{sub}, and ATSC3s indicates that the final equation is not an arbitrary one-off fit, even though the exact fold-specific symbolic form varies. At the same time, the lower recurrence of ASA, Xp_{7dv}, and MWC07 indicates that some components of Eq. 6 are more context-dependent than the recurrent core and should therefore be interpreted more cautiously.

References

(1) Tuttle, M. R.; Brackman, E. M.; Sorourifar, F.; Paulson, J.; Zhang, S. Predicting the Solubility of Organic Energy Storage Materials Based on Functional Group Identity and Substitution Pattern. *J. Phys. Chem. Lett.* **2023**, *14* (5), 1318–1325.

(2) Groom, C. R.; Bruno, I. J.; Lightfoot, M. P.; Ward, S. C. The Cambridge Structural Database. *Acta Crystallogr. Sect. B Struct. Sci. Cryst. Eng. Mater.* **2016**, *72* (2), 171–179.

(3) Bruno, I. J.; Cole, J. C.; Edgington, P. R.; Kessler, M.; Macrae, C. F.; McCabe, P.; Pearson, J.; Taylor, R. New Software for Searching the Cambridge Structural Database and Visualizing Crystal Structures. *Acta Crystallogr. Sect. B Struct. Sci.* **2002**, *58* (3), 389–397.

(4) Choudhary, K.; Tavazza, F. Convergence and Machine Learning Predictions of Monkhorst-Pack k-Points and Plane-Wave Cut-off in High-Throughput DFT Calculations. *Comput. Mater. Sci.* **2019**, *161* (15), 300–308.

(5) Acree, W.; Chickos, J. S. Phase Transition Enthalpy Measurements of Organic and Organometallic Compounds. Sublimation, Vaporization and Fusion Enthalpies From 1880 to 2015. Part 1. C1-C10. *J. Phys. Chem. Ref. Data* **2016**, *45* (3), 55.

(6) Acree, W.; Chickos, J. S. Phase Transition Enthalpy Measurements of Organic Compounds. An Update of Sublimation, Vaporization, and Fusion Enthalpies from 2016 to 2021. *J. Phys. Chem. Ref. Data* **2022**, *51* (4), 0.

(7) Prandini, G.; Marrazzo, A.; Castelli, I. E.; Mounet, N.; Marzari, N. Precision and Efficiency in Solid-State Pseudopotential Calculations. *npj Comput. Mater.* **2018**, *4* (1), 72.

(8) Hanwell, M. D.; Curtis, D. E.; Lonie, D. C.; Vandermeerschd, T.; Zurek, E.; Hutchison, G. R. Avogadro: An Advanced Semantic Chemical Editor, Visualization, and Analysis Platform. *J. Cheminform.* **2012**, *4* (8), 1–17.

(9) Hjorth Larsen, A.; JØrgen Mortensen, J.; Blomqvist, J.; Castelli, I. E.; Christensen, R.; Dułak, M.; Friis, J.; Groves, M. N.; Hammer, B.; Hargus, C.; Hermes, E. D.; Jennings, P. C.; Bjerre Jensen, P.; Kermode, J.; Kitchin, J. R.; Leonhard Kolsbjerg, E.; Kubal, J.; Kaasbjerg, K.; Lysgaard, S.; Bergmann Maronsson, J.; Maxson, T.; Olsen, T.; Pastewka, L.; Peterson, A.; Rostgaard, C.; SchiØtz, J.; Schütt, O.; Strange, M.; Thygesen, K. S.; Vegge, T.; Vilhelmsen, L.; Walter, M.; Zeng, Z.; Jacobsen, K. W. The Atomic Simulation Environment - A Python Library for Working with Atoms. *J. Phys. Condens. Matter* **2017**, *29* (27), 273002.

(10) MacRae, C. F.; Sovago, I.; Cottrell, S. J.; Galek, P. T. A.; McCabe, P.; Pidcock, E.; Platings, M.; Shields, G. P.; Stevens, J. S.; Towler, M.; Wood, P. A. Mercury 4.0: From Visualization to Analysis, Design and Prediction. *J. Appl. Crystallogr.* **2020**, *53* (1), 226–235.

(11) Gavezzotti, A.; Filippini, G. Theoretical Aspects and Computer Modeling of the Molecular Solid State; Gavezzotti, A., Ed.; Wiley and Sons: Chichester, 1998; pp 61–99.

(12) McDonagh, J. L.; Palmer, D. S.; Mourik, T. Van; Mitchell, J. B. O. Are the Sublimation Thermodynamics of Organic Molecules Predictable? *J. Chem. Inf. Model.* **2016**, *56* (11), 2162–2179.

(13) Landrum, G.; Tosco, P.; Kelley, B.; Rodriguez, R.; Cosgrove, D.; Vianello, R.; sriniker; Gedeck, P.; Jones, G.; Schneider, N.; Kawashima, E.; Nealschneider, D.; Dalke, A.; Swain, M.; Cole, B.; Turk, S.; Savelyev, A.; Vaucher, A.; Wójcikowski, M.; Take, I.; Tadhurst-cdd; Scalfani, V. F.; Walker, R.; Ujihara, K.; Probst, D.; Lehtivarjo, J.; Godin, G.; Pahl, A.; Berenger, F.; Faara, H.; Jasondbiggs; Strets123. RDKit: Open-Source Cheminformatics. 2024.

(14) Kier, L. B.; Hall, L. H. Derivation and Significance of Valence Molecular Connectivity. *J. Pharm. Sci.* **1981**, *70* (6), 583–589.

(15) Moriwaki, H.; Tian, Y. S.; Kawashita, N.; Takagi, T. *Mordred Chi*. Mordred 1.2.1a1 documentation.

(16) Gálvez, J.; Garcia, R.; Salabert, M. T.; Soler, R. Charge Indexes. New Topological Descriptors. *J. Chem. Inf. Comput. Sci.* **1994**, *34* (3), 520–525.

(17) Roy, K.; Ghosh, G. QSTR with Extended Topochemical Atom Indices. 2. Fish Toxicity of Substituted Benzenes. *J. Chem. Inf. Comput. Sci.* **2004**, *44* (2), 559–567.

(18) Moriwaki, H.; Tian, Y. S.; Kawashita, N.; Takagi, T. *Mordred Autocorrelation Module*. Mordred 0.3.1 documentation.

(19) Moriwaki, H.; Tian, Y. S.; Kawashita, N.; Takagi, T. *Mordred WalkCount*. Mordred 1.2.1a1 documentation.

(20) Labute, P. A Widely Applicable Set of Descriptors. *J. Mol. Graph. Model.* **2000**, *18* (4–5), 464–477.

Gaussian 16 Citation

Frisch, M. J.; Trucks, G. W.; Schlegel, H. B.; Scuseria, G. E.; Robb, M. A.; Cheeseman, J. R.; Scalmani, G.; Barone, V.; Petersson, G. A.; Nakatsuji, H.; Li, X.; Caricato, M.; Marenich, A. V.; Bloino, J.; Janesko, B. G.; Gomperts, R.; Mennucci, B.; Hratchian, H. P.; Ortiz, J. V.; Izmaylov, A. F.; Sonnenberg, J. L.; Williams–Young, D.; Ding, F.; Lipparini, F.; Egidi, F.; Goings, J.; Peng, B.; Petrone, A.; Henderson, T.; Ranasinghe, D.; Zakrzewski, V. G.; Gao, J.; Rega, N.; Zheng, G.; Liang, W.; Hada, M.; Ehara, M.; Toyota, K.; Fukuda, R.; Hasegawa, J.; Ishida, M.; Nakajima, T.; Honda, Y.; Kitao, O.; Nakai, H.; Vreven, T.; Throssell, K.; Montgomery, J. A., Jr.; Peralta, J. E.; Ogliaro, F.; Bearpark, M. J.; Heyd, J. J.; Brothers, E. N.; Kudin, K. N.; Staroverov, V. N.; Keith, T. A.; Kobayashi, R.; Normand, J.; Raghavachari, K.; Rendell, A. P.; Burant, J. C.; Iyengar, S. S.; Tomasi, J.; Cossi, M.; Millam, J. M.; Klene, M.; Adamo, C.; Cammi, R.; Ochterski, J. W.; Martin, R. L.; Morokuma, K.; Farkas, O.; Foresman, J. B.; Fox, D. J. Gaussian 16, Revision C.01, Gaussian, Inc., Wallingford CT, 2016
